# Improving iPSC Differentiation Using a Nanodot Platform

**DOI:** 10.1021/acsami.4c04451

**Published:** 2024-07-01

**Authors:** Men Yee Chiew, Erick Wang, Kuan-Chun Lan, Yan-Ren Lin, Yu-Huan Hsueh, Yuan-Kun Tu, Chu-Feng Liu, Po-Chun Chen, Huai-En Lu, Wen Liang Chen

**Affiliations:** † Center for Regenerative Medicine and Cellular Therapy, National Yang Ming Chiao Tung University, Hsinchu, 300, Taiwan, ROC; ‡ Department of Biological Science and Technology, National Yang Ming Chiao Tung University, Hsinchu 300, Taiwan, ROC; § College of Biological Science and Technology Industrial Ph. D. Program, National Yang Ming Chiao Tung University, Hsinchu 300, Taiwan, ROC; ∥ Center for iPS Cell Research and Application (CiRA), Kyoto University, Kyoto 606-8397, Japan; ⊥ Department of Emergency and Critical Care Medicine, Changhua Christian Hospital, Changhua 500, Taiwan, ROC; # Department of Post Baccalaureate Medicine, College of Medicine, National Chung Hsing University, Taichung 402, Taiwan, ROC; ¶ School of Medicine, Kaohsiung Medical University, Kaohsiung 807, Taiwan, ROC; ∇ School of Medicine, Chung Shan Medical University, Taichung 402, Taiwan, ROC; ○ College of Biological Science and Technology, National Yang Ming Chiao Tung University, Hsinchu 300, Taiwan, ROC; ⧫ Department of Orthopedic Surgery, E-Da Hospital, I-Shou University, Kaohsiung 824, Taiwan; †† Emergency Medicine Department, Kaohsiung Chang Gung Memorial Hospital, Kaohsiung, 833, Taiwan, ROC; ‡‡ Ph. D. Degree Program of Biomedical Science and Engineering, National Yang Ming Chiao Tung University, Hsinchu 300, Taiwan, ROC; §§ Institute of Materials Science and Engineering, National Taipei University of Technology, Taipei 106, Taiwan, ROC; ∥∥ Institute of Biochemistry and Molecular Biology, National Yang Ming Chiao Tung University, Hsinchu 300, Taiwan, ROC; ⊥⊥ Bioresource Collection and Research Center, Food Industry Research and Development Institute, Hsinchu City 300, Taiwan, ROC

**Keywords:** nanotopography, iPSC differentiation, differentiation efficiency, drug screening, cardiomyocyte differentiation mechanism

## Abstract

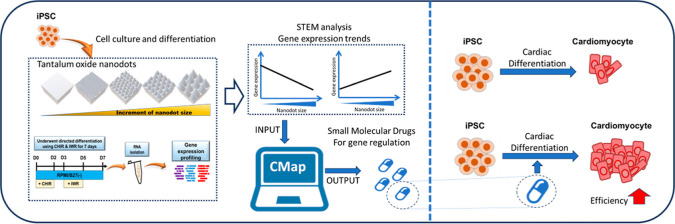

Differentiation of induced pluripotent stem cells (iPSCs) is an extremely complex process that has proven difficult to study. In this research, we utilized nanotopography to elucidate details regarding iPSC differentiation by developing a nanodot platform consisting of nanodot arrays of increasing diameter. Subjecting iPSCs cultured on the nanodot platform to a cardiomyocyte (CM) differentiation protocol revealed several significant gene expression profiles that were associated with poor differentiation. The observed expression trends were used to select existing small-molecule drugs capable of modulating differentiation efficiency. BRD K98 was repurposed to inhibit CM differentiation, while iPSCs treated with NSC-663284, carmofur, and KPT-330 all exhibited significant increases in not only CM marker expression but also spontaneous beating, suggesting improved CM differentiation. In addition, quantitative polymerase chain reaction was performed to determine the gene regulation responsible for modulating differentiation efficiency. Multiple genes involved in extracellular matrix remodeling were correlated with a CM differentiation efficiency, while genes involved in the cell cycle exhibited contrasting expression trends that warrant further studies. The results suggest that expression profiles determined via short time-series expression miner analysis of nanodot-cultured iPSC differentiation can not only reveal drugs capable of enhancing differentiation efficiency but also highlight crucial sets of genes related to processes such as extracellular matrix remodeling and the cell cycle that can be targeted for further investigation. Our findings confirm that the nanodot platform can be used to reveal complex mechanisms behind iPSC differentiation and could be an indispensable tool for optimizing iPSC technology for clinical applications.

## 1 Introduction

Induced pluripotent stem cells (iPSCs) possess unlimited self-renewal and the ability to differentiate into a wide variety of desired cell types^1^ and have been studied extensively for applications in biomedical fields, such as regenerative medicine,^2^ drug screening,^3^ and disease modeling.^4^ Despite this vast potential, however, practical implementation of iPSC technology is severely hindered by a poor understanding of the extremely complex differentiation process. iPSC differentiation is coordinated by continuous, elaborate interactions between an intricate ensemble of signaling pathways, many of which remain unclear.^5,6^ For example, though Wnt/β-catenin signaling has been well known to regulate cardiomyocyte (CM) differentiation in a biphasic manner,^7^ only recently have the temporal requirements of Wnt signaling been investigated.^8^ In addition, in vivo stem cell differentiation is heavily guided by the surrounding microenvironment or stem cell niche. While the stem cell niche provides critical physical and mechanical cues that regulate behaviors, such as proliferation, cell adhesion, and self-renewal, to modulate stem cell fate in vivo,^9^ this crucial regulation may not be accurately or consistently represented across various in vitro iPSC culture protocols.^10^ Discrepancies in iPSC behavior are further exacerbated by inherent dissimilarities between cells of different origin. Both genetic variability^11^ as well as parental cell type^12^ influence epigenetic and transcriptional profiles and can greatly affect iPSC performance. Together, these factors represent a major obstacle in comprehending and optimizing the differentiation process. As a result, complications, such as poor differentiation rate,^13^ differentiation into undesired cell types,^14^ and contamination with undifferentiated iPSCs leading to teratoma and malignant tumor formation,^15^ have been well documented. There is therefore an urgent need for a method of elucidating iPSC differentiation in order to maximize iPSC potential and achieve the consistency required for clinical applications.

Nanoengineering has recently emerged as a method for improving stem cell differentiation by providing external stimulation that normally guides differentiation in vivo. Numerous studies have demonstrated enhanced control over the stem cell behavior and fate in stem cells cultured on substrates with various nanoscale surface structures. For example, tantalum oxide (TiO_2_) nanotubes were shown to promote differentiation of mesenchymal stem cells into endothelial and smooth muscle cells.^16^ In addition, endocytosis of integrin receptors was enhanced by culturing cells on quartz nanopillars, thus reducing focal adhesion and cell stiffness.^17^ Moreover, aligned nanofibers were found to greatly promote migration of skin cells to improve wound healing.^18^ However, although culturing stem cells on substrates with nanostructures of a particular shape or size has yielded promising results, the mechanisms responsible remain unclear. Though many reports focus on the ability to enhance differentiation using nanotopography, few have analyzed the gene regulation responsible for the observed improvements, likely because the complexity of stem cell differentiation cannot be fully represented by any single nanotopography of a fixed dimension. Indeed, very few studies have explored the possibility of studying the differentiation process using nanomaterials. Our previous research demonstrates the ability to observe biological processes using nanotopographies of sequential size.^19^ By culturing cancer cells on sequential nanodot arrays of increasing size, we were able to visualize the epithelial–mesenchymal transition (EMT), a process linked to metastasis development, by exposing critical gene expression trends. Notably, we found that nanodot arrays acted as artificial microenvironments by simulating the mechanical cues normally provided by the cancer extracellular matrix (ECM) to encourage EMT. These results suggested the potential for using nanotopographies to study iPSC differentiation as well. While both physical and biochemical signaling are critical to successful stem cell differentiation, traditional methods of directing differentiation focus much more heavily on chemical activation of stem cells. Due to the critical role that the stem cell niche plays in guiding differentiation, we hypothesized that nanodot arrays acting as an artificial ECM would provide necessary physical stimulation that when combined with conventional differentiation protocols could reveal key gene expression trends related to iPSC differentiation that would be difficult to observe otherwise.

In this research, we observed the directed differentiation of iPSCs cultured on nanodot arrays with increasing diameters into CMs. Nanodot-induced gene expression changes were analyzed, allowing for the identification of small-molecule drugs that could potentially modify differentiation efficiency. Notably, time course treatments revealed that selected drugs could significantly enhance the cardiac differentiation efficiency of a cell line with inherently low CM differentiation propensity. Our results also provide additional insights into the transcriptional profiles responsible for enhanced differentiation. In summary, this study illustrates the utility and versatility of the nanodot platform as a tool for revealing mechanisms key to iPSC differentiation.

## 2 Materials and Methods

### 2.1 Fabrication of Nanodot Platform Consisting of Nanodot Arrays

Highly uniform nanodot arrays ranging from 10 to 200 nm were fabricated following the protocols from our previous study^19^ with slight modifications. A 200 nm thick tantalum layer was sputtered onto a 6 in. silicon wafer (Summit-Tech, West Hartford, CT, USA) followed by deposition of 400 nm thick aluminum onto the top of a tantalum (Ta) layer. The resulting aluminum TaN-coated Si wafers were then used to fabricate the nanodot arrays. Fabrication of the flat control substrate was done by dissolving the aluminum-coated wafer in 1 M NaOH before carrying out anodization in 0.3 M oxalic acid at 50 V/0.08 A for 10 min. To fabricate 10 nm nanodot arrays, anodization was carried out in 1.8 M sulfuric acid at 5 V/0.08 A for 90 min. For 50, 100, and 200 nm nanodots, a two-step anodization method was used. For 50 and 100 nm nanodot arrays, the first anodization step was carried out in 0.3 M oxalic acid at 25 V/0.4 A for 10 min and at 50 V/1.7 A for 20 min, respectively. For 200 nm nanodot arrays, anodization was performed in 5% (w/v) phosphoric acid at 120 V/0.13 A for 5 min. The porous alumina (50, 100, and 200 nm) was then removed by immersion in 5% (w/v) phosphoric acid for 40 min (50 nm surface), 70 min (100 nm surface), and 60 min (200 nm surface). Anodization was then repeated as described, but for 20 and 30 min for 10 and 50 nm nanodot arrays, respectively. Finally, porous anodic alumina was removed by immersion in 5% (w/v) phosphoric acid overnight.

### 2.2 Cold Field-Emission Scanning Electron Microscopy

To characterize the dimensions and homogeneity of nanodot arrays, substrates were coated with a thin layer of gold before measurement by field-emission scanning electron microscopy (FE-SEM) (HITACHI Regulus 8100). Images were captured with an accelerating voltage ranging from 5 to 10 kV. The diameters of nanodots from 6 batches were analyzed and measured using ImageJ.

### 2.3 Atomic Force Microscopy

The topography of nanodots was visualized using tapping mode atomic force microscopy (AFM) (Bruker Innova) with a scan area of 2 × 2 μm^2^ and analyzed with NanoScope Analysis software (Ver. 1.5).

### 2.4 Contact Angle Measurement

The wettability of nanodots was measured by using the Sessile and Captive Drop method with a video-based optical contact angle meter (model 100SB, Sindatek Instruments Corporation, Taipei, Taiwan). When 20 μL of deionized water was dropped and the nanodot surface was contacted, a snapshot was taken and the contact angle was calculated. Measurement was taken three times for each nanodot surface at the upper-left corner and center and bottom-right corners.

### 2.5 iPSC Lines and iPSC Culture

iPSC lines (T0104, I0303, and I0402) were provided by Food Industry Research and Development Institute (FIRDI), Taiwan, ROC. The cell lines were derived from human peripheral blood mononucleic cells using a Cytotune Sendai reprogramming kit (Invitrogen). Cells were expanded on a Matrigel hESC-Qualified Matrix, LDEV-free (Corning)-coated surfaces in a StemFlex medium (Thermo Fisher Scientific). The cells were maintained in a 37 °C, 5% carbon dioxide (CO_2_), and 95% humidity air incubator.

### 2.6 Immunofluorescence Staining

To perform immunofluorescence (IF) staining, cells were fixed with 4% paraformaldehyde for 15 min at room temperature (RT) followed by washing three times with phosphate-buffered saline (PBS). Cells were then permeabilized with 0.5% Triton X-100 in PBS for 10 min at RT. After washing with PBS, cells were blocked with 1% bovine serum albumin (BSA) in PBS for 1 h at RT before staining with primary antibodies (Abs) at 4 °C overnight. The next day, cells were washed and stained with secondary Abs for 1 h at RT in the dark. Cells were washed before being mounted with Prolong Diamond Antifade Mountant with DAPI (Invitrogen) and sealed on a glass slide. Images were captured using a Zeiss fluorescence microscope and visualized by AxioVision software. Dilution of primary and secondary Abs were used as follows: rabbit antihuman OCT4 (1:200), mouse antihuman SSEA4 (1:100), rat antihuman SOX2 (1:100), mouse antihuman TRA-1–60 (1:100); Alexa Fluor 594 donkey antirabbit (1:250), Alexa Fluor 488 goat antimouse (1:250), Alexa Fluor 488 donkey antirat (1:250), and Alexa Fluor 594 goat antimouse (1:250); primary mouse antihuman cTnT (1:100); primary rabbit antihuman α-actinin (1:250); secondary goat antimouse IgG conjugated Alexa Fluor 594 (1:1000); secondary donkey antirabbit conjugated Alexa Fluor 488 (1:1000); phalloidin conjugated Alexa Fluor 488 (1:400). All IF antibodies were purchased from Thermo Scientific.

### 2.7 Flow Cytometry

For flow cytometry, cells were harvested and resuspended at a concentration of 1 × 10^6^ cells/ml per tube. Cells were fixed and permeabilized with Cytofix/Cytoperm solution (BD Biosciences) for 30 min at 4 °C in the dark. Cells were washed with PBS two times before incubating antibodies or isotype control at 4 °C in the dark for 1 h. Cells were then washed and resuspended in PBS. For acquisition, the quantitative data of stained cells were acquired using BD FACSCantoII flow cytometry (BD Biosciences) and BD FACSDiva software (BD Biosciences). The data were analyzed and plotted by using FACS Express software (BD Biosciences). Antibodies for flow cytometry were purchased from BD Biosciences. Volumes of Abs usage was stated as following: 20 μL per reaction for PE mouse antihuman OCT4, PE mouse antihuman NANOG, PE mouse antihuman TRA-1–60, PE mouse antihuman TRA-1–81, and PE mouse antihuman SSEA4; 5 μL for PE mouse antihuman SOX2; and 5 μL per reaction for both PE mouse antihuman cTnT and PE mouse IgG1.

### 2.8 Differentiation of iPSC Lines into CMs

For CM differentiation, iPSCs with an 80% confluence were used. Cells were maintained in a 37 °C, 5% CO_2_ incubator with a RPMI 1640 medium (Thermo Fisher Scientific) as a basal medium. From day 0 to day 7, cells were cultured in a RPMI 1640 medium supplemented with 1 x B27 minus insulin (Thermo Fisher Scientific). Cells were exposed to the GSK3 -β inhibitor CHIR 99021 (6 μM; Selleckchem) from day 0 to day 1 followed by the Wnt antagonist IWR (5 μM; Sigma-Aldrich) from day 3 to day 5. From day 7 to day 21, cells were maintained in a RPMI 1640 medium supplemented with 1 x B27 (Thermo Fisher Scientific). For drug treatment experiments, T0104 iPSCs were treated with 1 μM carmofur, 0.1 μM KPT-330, 2.5 μM NSC 663284, or 10 μM BRD K98 from day 5 to day 7 of differentiation.

### 2.9 Differentiation of T0104 Cultured on Nanodot Arrays into CMs

Nanodot substrates were submerged in 75% alcohol for at least 2 h followed by 2 times washing with Milli-Q water. Substrates were thoroughly dried and then sterilized under UV light for at least half an hour before use. To culture T0104 on nanodot arrays, single cells were harvested using Accutase (StemCell Technologies). A total of 1.25 × 105/cm^2^ cells in 400 μL medium were seeded on each nanodot substrate (2 cm × 2 cm) and incubated in a 37 °C, 5% CO_2_ incubator for 5 h prior to adding additional 2 mL medium. The next day, cells were subjected to the differentiation protocol described in 2.5. After 7 days of differentiation, cells were harvested for characterization.

### 2.10 RNA Extraction

Total RNA was extracted using a TRIzol Reagent (Thermo Fisher Scientific) and Direct-zol RNA Purification kit (Zymo Research) according to the manufacturer’s protocols. Total RNA was eluted using DNase/RNase-free water and quantitated using a NanoDrop (Thermo Fisher Scientific) prior to subsequent assays. Integrity of the RNA was checked using Agilent TapeStation Systems (Agilent).

### 2.11 RNA Sequencing (RNA-Seq)

For RNA-seq, high-throughput next-generation sequencing was performed. The RNA library was constructed according to the manufacturer’s protocol. In brief, poly-A tail RNA was isolated, followed by fragmentation of RNA. The fragmented poly-A RNA was then reverse transcribed into cDNA using random primers. The cDNA was adenylated at the 3′ end prior to adapter ligation. The adapter ligated cDNA was then polymerase chain reaction (PCR) amplified to obtain larger amounts of the cDNA library. The quantity of the cDNA library was measured by real-time PCR and Qubit fluorometry (Invitrogen). The size of the cDNA library was measured using an Agilent D1000 ScreenTape System (Agilent). The validated cDNA was then sequenced using a Hi-Seq sequencer (Illumina) according to the standard workflow.

### 2.12 RNA-Seq Analysis

Raw sequencing data were processed with fastp (Ver.0.21.0) for quality profiling, adapter trimming, read filtering, and base correction.^20^ The quality of preprocessed data was then checked and visualized using FastQC (Ver.0.11.9) and MultiQC (Ver.1.10.1).^21,22^ The QC-passed reads were aligned to the human reference genome (GENCODE GRCh38 v38)^23^ using STAR (Ver.2.7.9a)^24^ two-pass mode mapping prior to quantification of the aligned reads using RSEM (Ver.1.3.3).^25^ The gene expression levels in transcripts per million (TPM) were then used for subsequent analysis.

### 2.13 Short-Time Series Expression Miner Analysis

Short time-series expression miner (STEM) (Ver.1.3.13)^26^ was used to identify significant gene expression patterns induced by different sizes of nanodots. Prior to analysis, genes with duplicated Ensembl gene IDs (ENSG_ID) on pseudoautosomal regions of chromosome Y (PAR_Y) and those genes with average TPM expression values less than 10 were removed. Then, STEM analysis was performed using the expression data of the 7686 remaining genes from 5 samples (flat, 10, 50, 100, and 200 nm nanodots). For settings, gene expression data was specified as log2 format (flat sample was defined as time point 0), the clustering method was set to STEM clustering with the maximum number of model profiles as 25 (default: 50), and the maximum unit change in model profiles between time points was set to 2 (default). For advanced options, maximum number of missing values was set to 4 and the minimum absolute expression change based on “Maximum–Minimum” was set to 1 (2-fold). All other advanced options were set to the default.

### 2.14 Connectivity Map Analysis

To identify potential small-molecule compounds related to the regulation of gene expression profiles induced by nanodot stimulation, we performed connectivity map (CMap) analysis (data version Beta, software version 1.2 build 1.44) through the “clue.io” cloud-based software platform (CMap and LINCS Unified Environment, CLUE).^27,28^ Genes selected from the statistically significant model profiles identified in STEM analysis were first converted from the Ensembl gene id to Entrez gene id using “g:convert” in g:Profiler,^29^ a web-based toolset, and the invalid genes were manually corrected. The “Query” web tool in CLUE platform was then used for the CMap analysis with the following parameters: “Gene expression (L1000)” assay, “Touchstone” reference and “Individual query” mode.

### 2.15 Reverse Transcription-Quantitative Polymerase Chain Reaction

Reverse transcription was carried out using Maxima First Strand cDNA Synthesis Kit for reverse transcription-quantitative polymerase chain reaction (Thermo Scientific, USA) according to the manufacturer’s protocol. Quantitative PCR was performed using a Taqman Gene Expression Assay (Applied Biosystems, USA) and Taqman Fast Advanced Master Mix (Applied Biosystems, USA) following the provided protocols. The PCR reaction was performed using the StepOnePlus Real-Time PCR System (Applied Biosystems, USA) The list of Taqman Gene Expression Assays and their ID is tabulated in Table S1.

### 2.16 Statistical Analysis

GraphPad Prism 7.04 software was used for statistical analysis. All data are represented as the mean ± standard deviation. One-way analysis of variance (ANOVA), *t*-test, and Sidak’s test were applied to calculate the differences between the distinct values. Values of *p* < 0.05 were considered statistically significant, presented as * = *p* < 0.05; ** = *p* < 0.005; *** = *p* < 0.0005; ****p < 0.0001.

## 3 Results and Discussion

### 3.1 Fabricated Nanodot Substrates Possess Well-Defined and Homogeneous Topographies Suitable for iPSC Culture

Recent reports have demonstrated the ability to influence various cellular processes by culturing cells on nanotopographical substrates with varying physical features, such as stiffness, size, and orientation.^30−32^ Tantalum pentoxide in particular is a biocompatible metal that has been extensively engineered on silicon substrates into nanotopographies of various sizes and shapes to regulate cell behaviors while avoiding inflammation or rejection of biological tissues.^33,34^ Our previous works have shown the ability of nanodot arrays to act as artificial tumor microenvironments to regulate focal adhesion and cellular transport.^35,36^ In breast cancer cells specifically, we demonstrated that larger nanodot diameter-modulated expression of key cell junction genes, including E-cadherin and N-cadherin to promote elongated and spindle-like cell shape as well as upregulation of EMT-related transcription factors Twist and Snail. Furthermore, nanodot arrays of increasing diameter between 10 and 200 nm were used to induce progressive stages of cancer metastasis simultaneously in lung cancer cells, thus enabling the visualization of the process of metastasis development.^19^ Importantly, the nanodots promoted EMT by providing physical cues normally provided by cancer ECM. Based on these findings, we hypothesized that TaOx nanodot arrays between 10 and 200 nm would serve as a suitable platform for studying the mechanisms involved in differentiation, a process that is also heavily dependent on mechanical signals provided by the stem cell niche.

**Figure 1:**
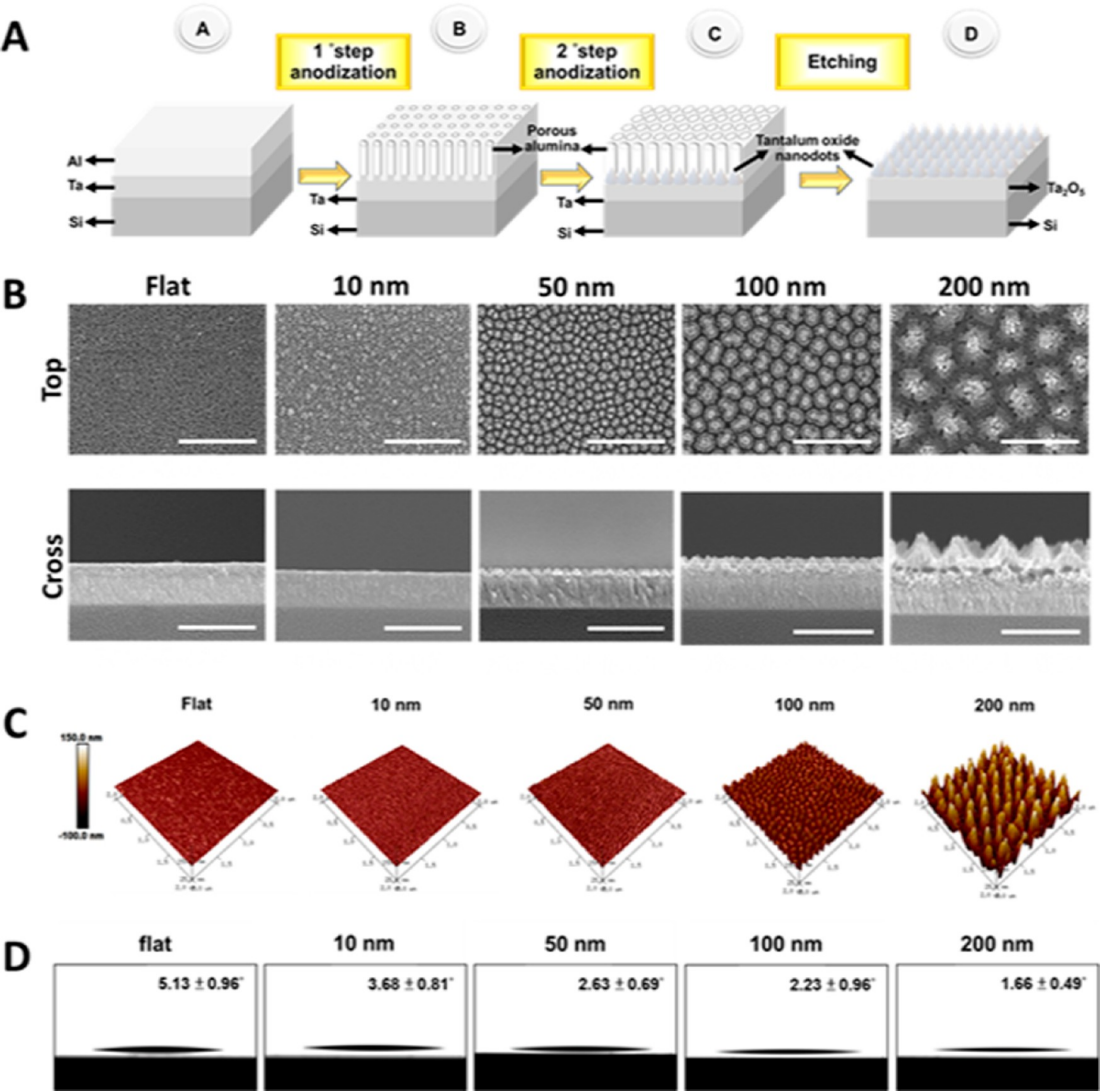
Nanodot arrays of increasing diameter are fabricated to form a nanodot platform. (A) Schematic diagram of fabrication of the TaOx nanodot arrays. (B) Top view and cross view SEM images of nanodot arrays. (C) AFM images of nanodot arrays. (D) Contact angle of nanodot arrays. Images are arranged from left to right: Flat, 10, 50, 100, and 200 nm. Scale bar = 500 nm.

To this end, four nanodot array surfaces composed of 10, 50, 100, and 200 nm diameter nanodots were produced. Additionally, a flat unpatterned TaOx surface was used as a control. One or two-step anodization was carried out to fabricate different sized nanodot arrays as described previously^19^ (Figure 1A). Cold field-emission SEM revealed dense parallel nanodot arrays (Figure 1B). AFM further confirms the well-defined conical nanodot shape and formation of organized arrays (Figure 1C). Diameters of 10, 50, 100, and 200 nm nanodot arrays were 10.22 ± 1.59 nm, 64.82 nm ± 5.36, 111.14 nm ± 7.98, and 235 nm ± 14.66, respectively (Figure S2). Additionally, contact angle of flat, 10, 50, 100, and 200 nm nanodots were 5.13 ± 0.96, 3.68 ± 0.81, 2.63 ± 0.69, 2.23 ± 0.96, and 1.66° ± 0.49, respectively (Figure 1D), indicating hydrophilicity appropriate for efficient cell attachment^37^ in all nanotopographies. Together, these results suggest consistent fabrication of differently sized nanodot arrays suitable for iPSC culture.

### 3.2 iPSCs Derived from Different Donors Exhibit Varying CM Differentiation Propensity

The highly variable differentiation efficiency between different iPSC lines represents a major obstacle in stem cell technology. To demonstrate this, three iPSC lines, T0104, I0402, and I0303, generated from three different donors were used in this study.

**Figure 2:**
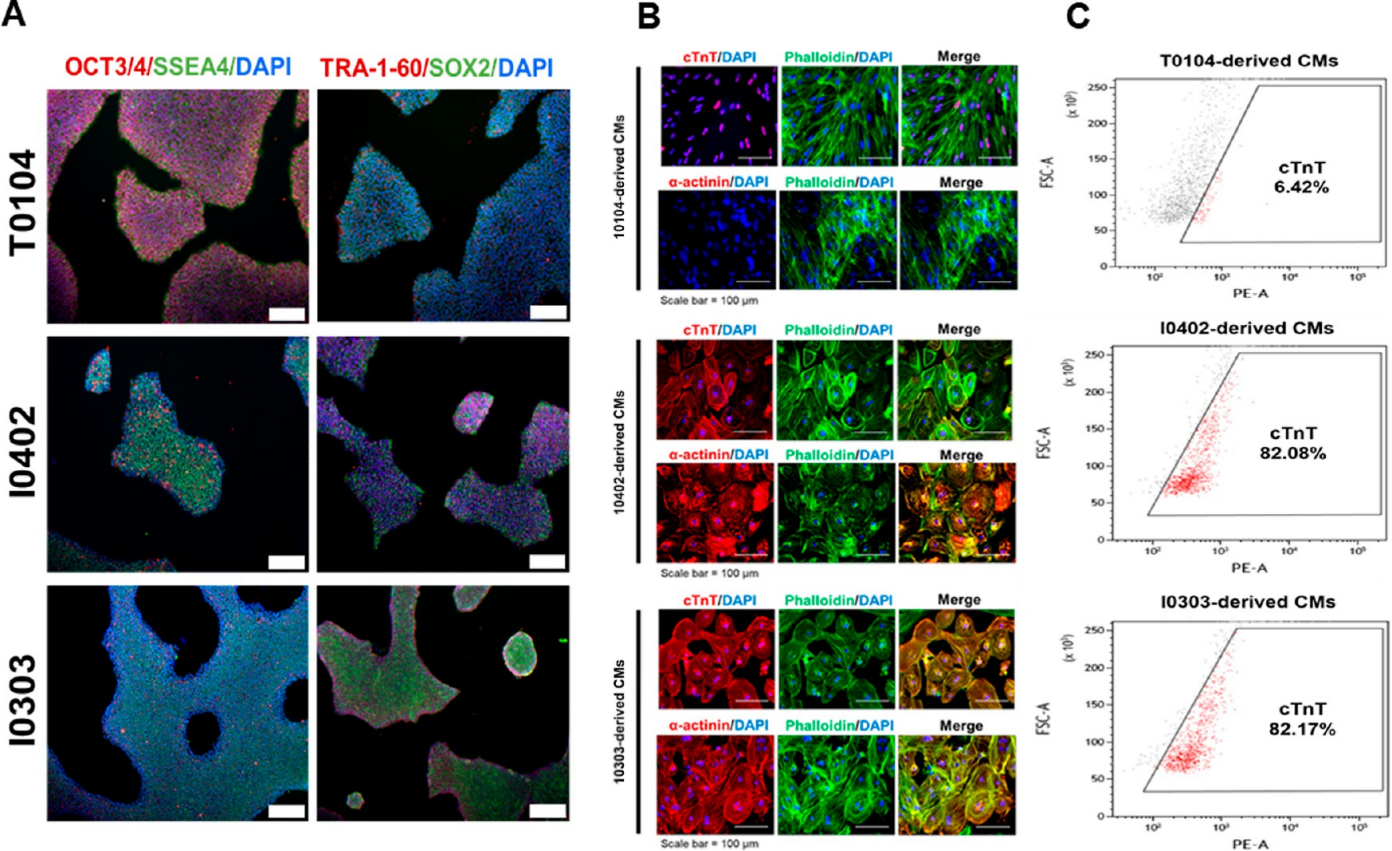
iPSCs derived from different sources exhibit variable differentiation efficiency. (A) IF staining of pluripotency markers OCT4, SOX2, TRA-1–60, and SSEA4 in T0104, I0303, and I0402 iPSC lines. (B) IF staining against CM markers cTnT and α-actinin, along with F-actin and nuclear DNA in T0104, I0303, and I0402-derived CMs after 21 days. (C) cTnT expression in T0104, I0303, and I0402-derived CMs after 21 days. Scale bar = 100 μm.

First, stem cell integrity was verified via the immunofluorescence staining of essential pluripotency markers. As shown in Figure 2A, both surface pluripotency markers (*SSEA4* and *TRA-1–60*) as well as stem cell-associated transcription factors (*OCT4* and *SOX2*) are clearly present in all three cell lines, confirming iPSC stemness.^38,39^ Once stem cell pluripotency was characterized, differentiation potential of each cell line was investigated by using the CHIR/IWR differentiation protocol^8^ to induce CM differentiation. Upon completion of protocol, IF staining of the cardiac muscle cell markers cardiac troponin-T (cTnT) and α-actinin^40^ was performed (Figure 2B). After 21 days, a noticeable difference in the morphology between cells of different origins was observed. T0104-derived cells became significantly extended, whereas I0402 and I0303-derived cells exhibited a much rounder morphology, suggesting a divergence in the differentiation process. More importantly, while cTnT was present in all three cell lines, expression levels were obviously inconsistent. While I0402- and I0303-derived CMs displayed prominent cTnT staining throughout the cytoplasm, T0104 exhibited a much lower cTnT expression, which seemed to be localized to the nuclei. Flow cytometry (Figure 2C) confirmed these results, indicating 82.08 and 82.17% cTnT expressions in I0402- and I0303-derived CMs, respectively, but only 6.42% expression in T0104-derived CMs. Notably, despite receiving the same differentiation protocol, the three cell lines showed varying differentiation potential. Namely, the low cardiac muscle marker expression levels of T0104 suggest poor differentiation efficiency when compared with both I0402 and I0303 cells. Similar results have been shown in other studies comparing differentiation propensity between iPSC lines of different origin.^41^ While immunofluorescence staining clearly demonstrates the difference in differentiation ability between the different cell lines, the mechanisms responsible for the apparent discrepancies are unclear. Namely, the transcriptional modulation resulting in well-differentiated I0402- and I0303-derived CMs but poorly differentiated T0104-derived CMs cannot be revealed by staining results alone. Because nanotopographies have demonstrated potential for revealing the mechanisms behind biological processes, T0104 was selected for further study via culturing on the fabricated nanodot platform.

### 3.3 iPSCs Cultured on a Nanodot Platform Reveal Gene Expression Trends Potentially Involved in the Differentiation Process

**Figure 3:**
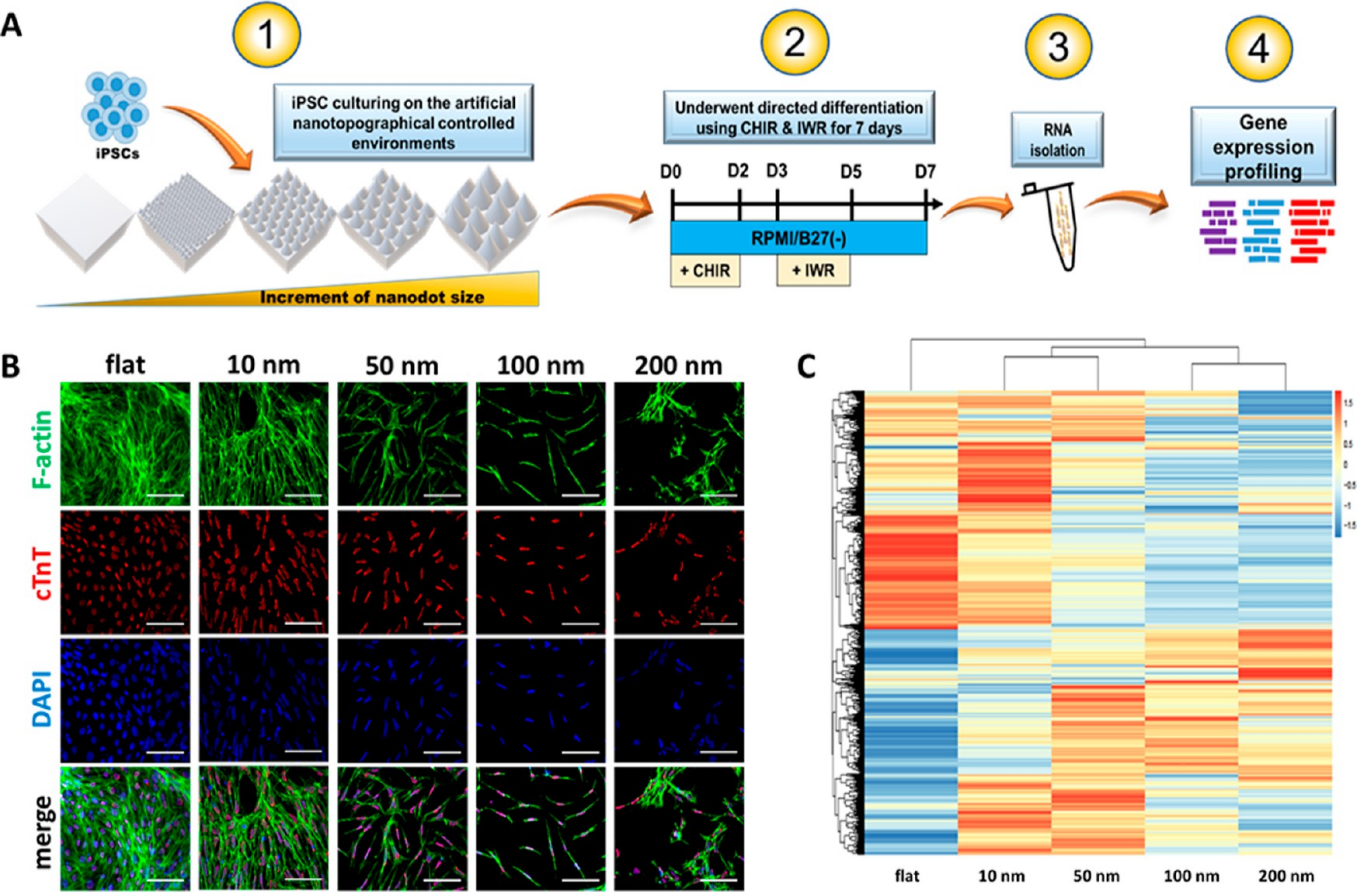
Nanodot platform reveals gene expression trends potentially related to low differentiation propensity exhibited by T0104 iPSCs. (A) Scheme illustrating the process of examining differentiation-related gene expression trends using the nanodot platform. (B) IF staining of cTnT, F-actin, and nuclear DNA in T0104-derived CMs after 7 days. (C) Heatmap showing changes in expression levels of genes in T0104-derived CMs as a nanodot diameter increases.

While the nanodot platform exhibited clear utility in the investigation of cancer dynamics, its applicability to iPSC-related studies has not yet been explored. Notably, in contrast to the induction of EMT in cancer cells in our previous work, which was triggered solely through physical stimulation via culturing on nanodot arrays, stem cell differentiation is normally regulated by both biochemical and physical signaling. For iPSCs, chemical induction of differentiation is achieved by drug treatment, while physical regulation provided by materials, such as Matrigel is often limited. The purpose of this work is to determine whether the combination of nanodot arrays with conventional chemical-based differentiation protocols could reveal additional insights regarding the mechanisms of differentiation. Specifically, because stem cell differentiation is tightly controlled by physical cues arising from the surrounding microenvironment, we hypothesized that the nanodot platform could serve as a more robust artificial stem cell niche and that differentiating iPSCs on the platform would reveal the overall stepwise transcriptional changes that are involved in the differentiation process in greater detail. To this end, T0104 cells were cultured on the fabricated nanodots in conjunction with a differentiation protocol, then harvested for gene expression profiling and IF staining according to the schedule shown in Figure 3A to monitor differentiation and identify any related changes in the gene expression.

IF staining of cells cultured on the nanodot platform is shown in Figure 3B. While the cTnT expression was observed, staining revealed that the expressed protein was limited to cell nuclei as opposed to being secreted to the cytoplasm, indicating poor differentiation. RNA sequencing of iPSCs cultured on different sized nanodots were organized into the heatmap, as shown in Figure 3C. The heatmap revealed two general changes in the gene expression that were induced by culture on the nanodot platform. Specifically, a majority of the genes in the top half were seemingly downregulated as the nanodot diameter increased, while many genes in the bottom half seemed to be upregulated by an increase in the nanodot size.

**Figure 4:**
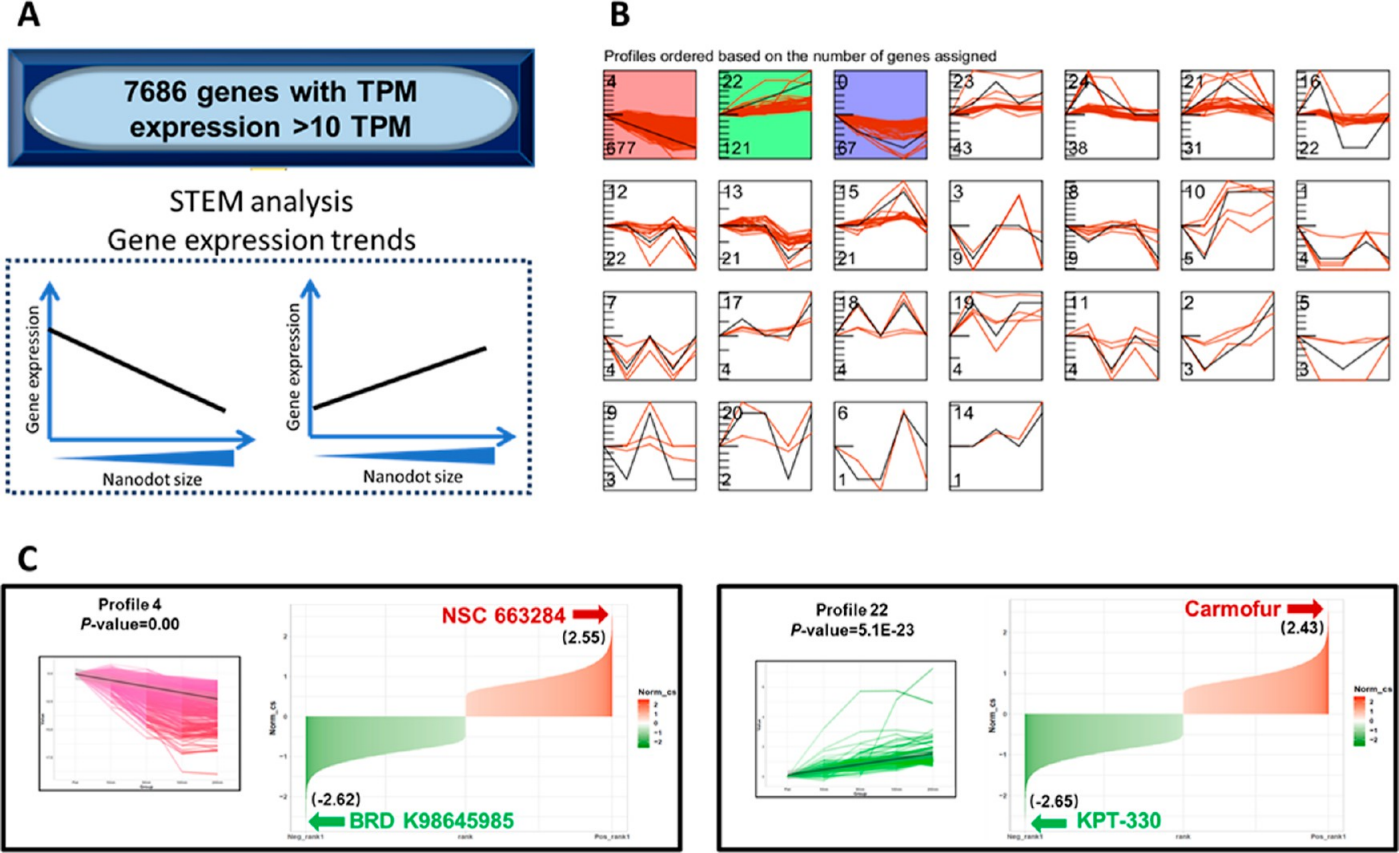
STEM-analysis reveals expression profiles significantly correlated with poor differentiation of T0104 that are used for drug screening via CMap. (A) Scheme illustrating gene selection requirement. Only genes with TPM >10 were selected for subsequent STEM analysis. (B) STEM model profiles of different gene expression patterns. Profiles are ordered based on the number gene assigned, and backgrounds of significant profiles are shaded. Scale bar = 100 μm. (C) STEM-determined significant profile 4 along with CMap-selected drugs BRD K98 (similar) and NSC-663284 (opposing) and STEM-determined significant profile 22 along with CMap-selected drugs carmofur (similar) and KPT-330 (opposing). BRD K98 shows negative connectivity score (cs) because gene expression trends across nanodot arrays were input in reverse for profile 4; NSC-663284 exhibits positive cs for the same reason.

To explore these results further and possibly identify significant gene expression trends responsible for the observed low differentiation efficiency, we applied STEM. Initially designed to analyze time series data, STEM has proven to be a useful tool for recognizing significant trends in data sets that have been sequentially ordered (i.e., gene expression of cells cultured on increment nanodot arrays).^26^ STEM categorized the 7876 genes into 25 unique expression profiles, or patterns, as shown in Figure 4B. Profiles 4, 22, and 9 were significantly correlated with an increase in the nanodot diameter (denoted by colored background in Figure 4B). The 677 genes in profile 4 were generally downregulated by the increased nanodot size, while the 121 genes in profile 22 were largely upregulated. Expression of the 67 genes in profile 9 initially decreased with the nanodot size, but increased halfway through, resulting in similar expression levels between cells cultured on flat vs 200 nm nanodots. Genes belonging to these three profiles were hypothesized to play an important role in causing the low differentiation ability of the T0104 iPSCs observed in Figure 3. In particular, profiles 4 and 22 demonstrate clear consistent trends in the gene expression and were selected for further investigation. Gene ontology (GO) term analysis was performed on the genes of profiles 4 and 22 to determine which biological processes they regulate (Figure S3). The expression levels of the 10 genes most significantly regulated by nanodot diameter from each profile are shown in Figure S4.

According to GO analysis results, genes in profile 4 play a major role in regulating the ECM as well as the cell shape and cell adhesion. The in vivo ECM is composed of a mesh of structural proteins including laminins and collagens and represents a critical component of the stem cell niche.^42^ The proteins that form the ECM possess a wide variety of physical and biochemical properties, thus conferring different biophysical and biochemical properties to the ECM depending on amount and composition.^43^ Modulation of the production, remodeling, and breakdown of ECM proteins alters key ECM attributes, such as topography, rigidity, and growth factor presentation, to provide crucial biochemical and mechanical cues that guide essential stem cell behaviors, such as stem cell renewal and differentiation.^44,45^ Cell adhesion and cell shape are also highly influenced by ECM remodeling, responding to changes in ECM stiffness via mechanotransduction to facilitate various stem cell behaviors.^46^ Many of the top 10 genes of profile 4 are related to ECM remodeling (*MMP9*), cell adhesion (*IGFBP7*, *CLDN7*), or cell shape (*KRT7*, *KRT19*). Notably, these genes were all down-regulated by increased nanodot diameter and correlated with poor differentiation efficiency, thus presenting an opportunity for potentially improving differentiation through targeted drug treatment.

Genes in profile 22 were found to be related to cell growth and the cell cycle. The association between changes in cell cycle progression and stem cell differentiation has been widely documented.^47,48^ In almost all stem cells, the initiation of differentiation is closely correlated with an irreversible arrest of the cell cycle that is mediated by upregulation of CDK inhibitor proteins (CKIs) and activation of retinoblastoma (Rb) proteins.^49^ Cyclin D1, a key regulator of G1 phase length, has been shown to play an integral role in recruiting transcriptional corepressors to facilitate differentiation into various germ layers.^50^ Additionally, mechanisms specific to the S and G2 phases were found to maintain stem cell pluripotency independently of G1 signaling pathways,^51^ further substantiating the critical function of cell cycle progression in regulating stem cell differentiation. Poor differentiation efficiency of cardiac cells on nanodots of increasing size was concomitant with the significant upregulation of many cell-growth-related genes in profile 22 (*VTRNA2*, *H4C12*, *RARB*, and *SFRP2*), and modulating expression of these genes may be a possible avenue for improving differentiation efficiency.

To explore the potential for improving cardiac differentiation in T0104, we utilized CMap, a repository that matches gene signatures with mechanism of action of small-molecule drugs.^27^ Specifically, we theorized that a perturbagen that could induce similar gene expression trends seen in profiles 4 and 22 could possibly interfere with directed differentiation of T0104 iPSCs even when cells are cultured following a normal protocol without nanodots. More importantly, the reverse could also be true: namely, a drug that modulates expression of the significant genes in an opposite manner could potentially improve the differentiation ability of T0104. To test this hypothesis, profiles 4 and 22 were input into the CMap database for the selection of drugs that had similar or opposing signatures to each profile. Query results were presented as a list of small molecular compounds ordered by a normalized connectivity score (norm-cs) (Tables S4–S7), with the more positive norm-cs indicating a more similar signature and vice versa. Figure 4C shows the similar and opposing drugs chosen for profile 4. Because CMap only allows positive trending inputs, gene expression data from profile 4 was entered in reverse sequence (i.e., from 200 nm to flat), and results were also interpreted in reverse. Of all drugs screened, BRD K98 had the lowest norm-cs, and therefore the most similar signature, to profile 4, and is therefore expected to reduce differentiation efficiency. In contrast, NSC-663284 had the highest norm-cs (opposing signature) and is expected to improve differentiation. Because genes in profile 22 naturally demonstrated an upregulated pattern, they were input into CMap without changing the order, and results were directly interpreted (Figure 4C). Carmofur (high norm-cs) showed the most similar signature, and KPT-330 (low norm-cs) was chosen as the opposite signature. The selected small-molecule drugs were then used in conjunction with standard cardiac differentiation protocols for experimental validation.

### 3.4 Small-Molecule Drugs Selected via CMap Can Significantly Improve Differentiation Efficiency

**Figure 5:**
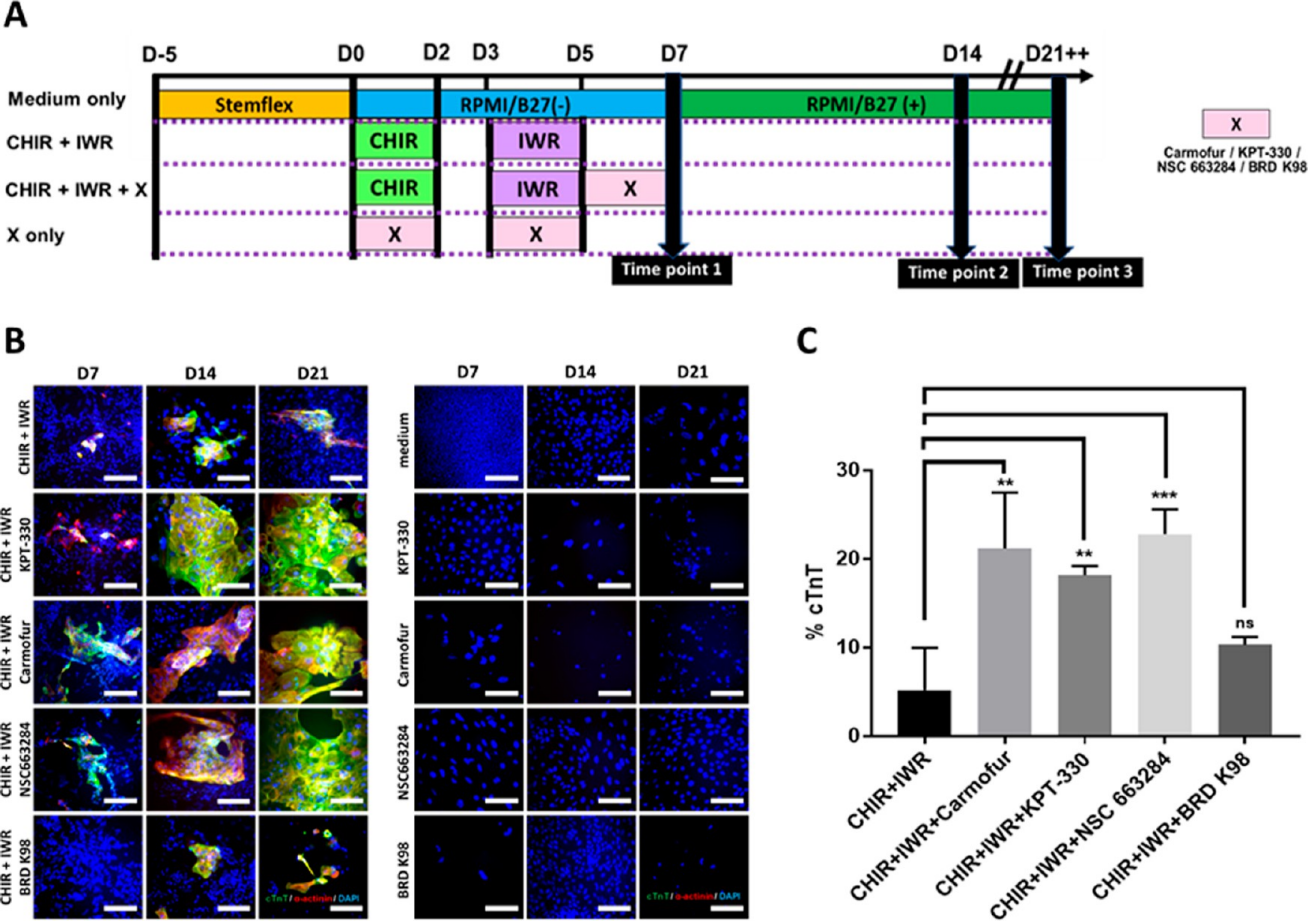
Small-molecule drug treatment successfully modulates differentiation efficiency of T0104 iPSCs. (A) Schedule for standard differentiation of T0104 iPSCs in conjunction with small-molecule drug treatment. (B) IF staining of cTnT, α-actinin, and nucleic DNA of T0104-derived CMs treated with various small-molecule drugs after 7, 14, and 21 days. (C) cTnT expression levels in T0104-derived CMs treated with various small-molecule drugs after 21 days (one-way ANOVA) (*p < 0.05, **p < 0.005, ***p < 0.0005, ****p < 0.0001). Scale bar = 100 μm.

**Figure 6:**
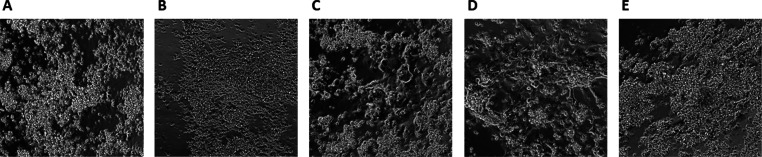
Small-molecule drug treatment successfully modulates the morphology and function of T0104-derived CMs.

The small-molecule drugs identified using CMap were used to treat iPSCs during standard CM differentiation according to the schedule shown in Figure 5A to observe their effects on the differentiation efficiency. IF staining was performed to assess α-actinin and cTnT expressions (Figure 5B). In addition, the cTnT expression was quantified by using flow cytometry (Figure 5C). Cells were also recorded under brightfield to record CM beating (Videos S1–S5). Stills of the videos are shown in Figure 6.

iPSCs treated with a standard differentiation protocol served as the control group. As expected, α-actinin and cTnT expression levels were low throughout the 21 day culturing period. After 21 days, the cTnT expression remained low at 5.2%. Furthermore, spontaneous beating was only observed in a small clump of cells after 21 days (Video S1).

BRD K98 and NSC-663284 were selected as similar and opposing drugs for profile 4, respectively. BRD K98 treatment resulted in poor CM differentiation. Notably, staining of BRD K98-treated iPSCs showed seemingly lower α-actinin and cTnT expression after both 7 and 14 days of differentiation when compared to the control group. Flow cytometry results showed 10.3% cTnT expression, which did not differ significantly from the expression in control cells after 21 days. Additionally, BRD K98-treated cells seemed to exhibit no beating at all after 21 days of differentiation (Video S2), indicating worse overall differentiation than control group. In contrast, NSC-663284 treatment-induced upregulation of both α-actinin and cTnT expressions, which could be clearly seen after 7, 14, and 21 days of differentiation via IF staining. Flow cytometry confirmed a significantly higher cTnT expression in NSC-663284-treated iPSCs (22.8%) than in control cells. Beating was observed in a much larger area of cell clumps as well (Video S3), suggesting increased cardiac function.

For profile 22, carmofur and KPT-330 were chosen as similar and opposing signature drugs, respectively. Surprisingly, though carmofur showed high similarity to the nanodot-induced profile 22 and was expected to inhibit differentiation, iPSCs treated with carmofur displayed markedly higher differentiation efficiency. Carmofur-treated iPSCs displayed clear expression of both α-actinin and cTnT after only 7 days, which increased considerably after 14 and 21 days. The cTnT expression was significantly higher in cells treated with carmofur (21.2%) compared with control cells. In addition, a noticeably larger area of cell clumps demonstrated beating after treatment with carmofur (Video S4). Thus, carmofur greatly improved the iPSC differentiation ability, challenging initial expectations. KPT-330 possessed an opposite signature from profile 22 and was predicted to enhance iPSC differentiation. Staining showed an obvious increase in expression of α-actinin and cTnT after treatment with KPT-330, and cTnT expression after 21 days was significantly higher in KPT-330-treated cells than in control cells. Additionally, the majority of cell clumps treated with KPT-330 exhibited beating (Video S5), indicating a greater differentiation efficiency.

Taken together, the results in Figure 5 and Videos S1–S5 confirm that the iPSC differentiation ability can be modified using small-molecule drugs identified via CMap. Particularly, differentiation of T0104 iPSCs was either inhibited or enhanced by the drugs selected based on the gene expression profiles revealed through culturing on the nanodot platform. BRD-K98, which regulates the gene expression in a manner similar to the nanodot arrays, was shown to further inhibit cardiac differentiation, as evidenced by the lack of beating in cell clumps. Both NSC-663284 and KPT-330, which possessed gene signatures that oppose nanodot regulation, improved expression of cardiac-specific markers as well as beating in cell clumps significantly.

Morphology and function of T0104-derived CMs under a bright-field microscope following treatment with the CHIR/IWR protocol only (A) or in conjunction with BRD K98 (B), NSC-663284 (C), carmofur (D), or KPT-330 (E). Videos recording the beating of clumps of CMs can be viewed via the HTML link.

### 3.5 Nanodot Platform Unveils Potentially Critical Genes to Facilitate Drug Screening and Provide Further Insights regarding CM Differentiation

**Figure 7:**
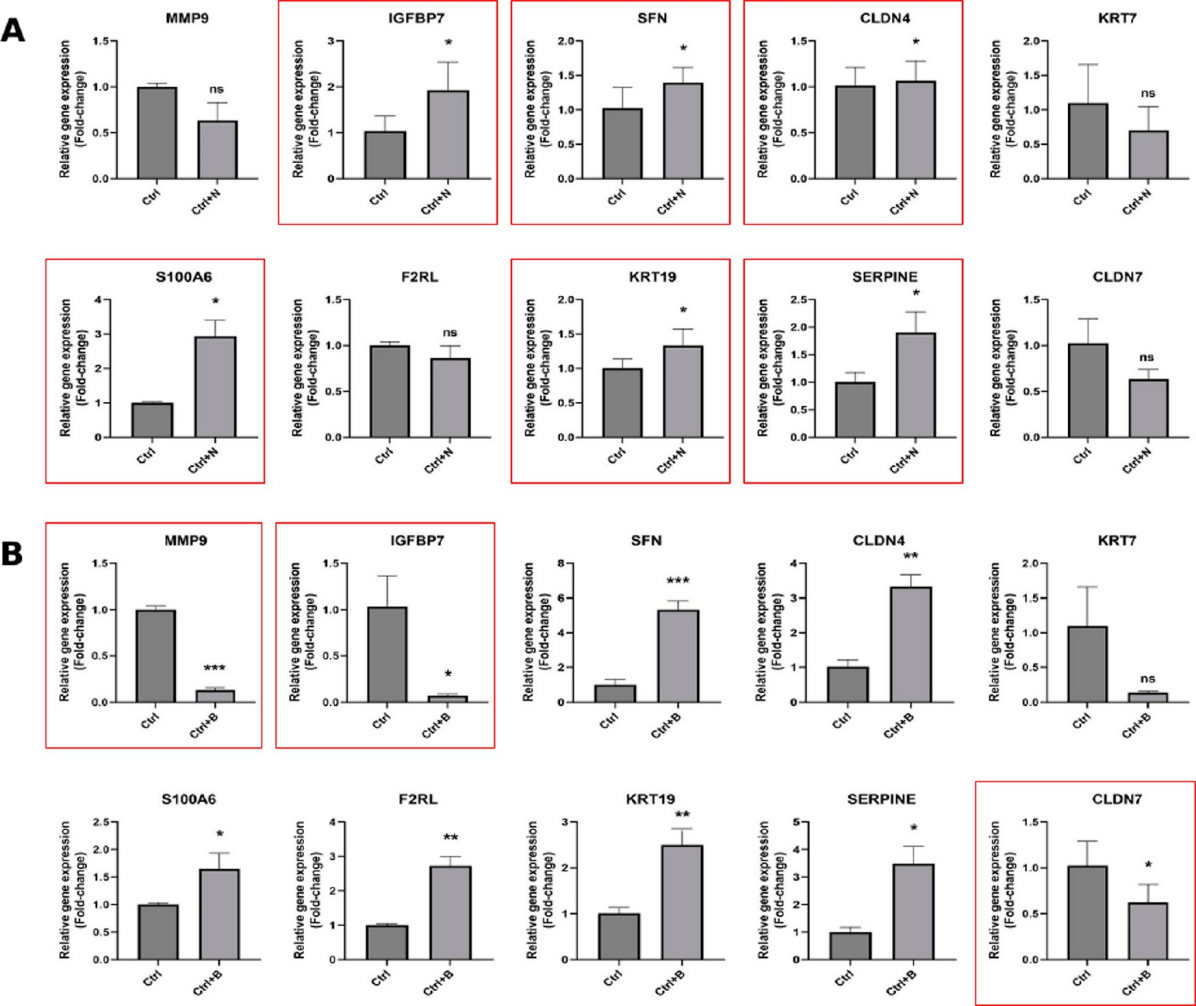
ECM-related genes of profile 4 are significantly modulated by treatment with CMap-selected small-molecule drugs.

Expression levels of 10 highly significant genes of profiles 4 and 22 were analyzed using quantitative polymerase chain reaction (qPCR) to determine the changes in gene regulation responsible for the differences in the differentiation efficiency following small-molecule drug treatment. Results for profile 4, which contains genes that regulate ECM, cell adhesion, and cytoskeleton, are shown in Figure 7. Four of the ten genes (*IGFBP7*, *S100A6*, *KRT19*, and *SERPINE*) were significantly upregulated after treatment with NSC-663284 (Figure 7A), while three of the ten genes (*MMP9*, *IGFBP7*, and *FN1*) were significantly downregulated after treatment with BRD K98 (Figure 7B). This bidirectional regulation of certain genes can likely at least partially explain the drastic change in the differentiation efficiency. For example, integrin-like growth factor-binding protein 7 (IGFBP7) was highly expressed in highly differentiated NSC-663284-treated cells but was significantly under-expressed in poorly differentiated BRD K98-treated cells. IGFPB7 was reported to promote migration in both glioma and pulmonary alveolar epithelial cells via activation of extracellular-signal-regulated kinase (ERK) pathway.^52,53^ Furthermore, overexpression of IGFBP-7 has been shown to activate expression of Runt-related transcription factor 2 (*RUNX2*) and *SP7*, the two master transcription factors that regulate osteogenesis, to enhance osteogenic differentiation of bone marrow-derived mesenchymal stem cells.^54^ More recently, inhibition of IGFBP7 expression was shown to partially prevent differentiation of ESCs into CMs.^55^ Our results align with these findings, further validating the role that IGFBP7 plays in CM differentiation specifically. Additionally, fibronectin (FN1) was also significantly downregulated by treatment with BRD K98 but not by the NSC-663284 treatment. FN1 is well-documented as a vital component of the ECM that mediates healing and remodeling in cardiac cells.^56^ Substrates comprised of FN1 and laminin were found to greatly improve differentiation of human epithelial stem cells into CMs by activating integrin β5 signaling,^57^ while knockdown of *FN1* has been shown to prevent mesoderm formation and subsequent CM differentiation in human pluripotent stem cells.^58^ The importance of *FN1* in regulating the early stages of CM differentiation and its significant downregulation in BRD K98-treated cells may explain the observed poor differentiation. On the other hand, *SERPINE* was one of the genes significantly upregulated by both NSC-663284 as well as BRD K98. Plasminogen activator inhibitor-1 (PAI-1), encoded by the *SERPINE* gene, is strongly implicated in myocardial proliferation and endocardial maturation^59^ and may play a role in CM differentiation by regulating myocardial remodeling.^60^ Indeed, PAI-1 deficient hiPSC-CMs were shown to exhibit much higher susceptibility to CM injury.^61^ Despite its pivotal role in myocardial proliferation, however, the upregulated expression of PAI-1 alone was unable to stimulate cardiac differentiation in iPSCs treated with BRD K98. Likewise, genes, such as *MMP9* and *KRT19*, known to be involved in the differentiation of multiple lineages,^62,63^ exhibit similar directional changes in the expression between the two treatment groups despite the obvious discrepancy in differentiation efficiency. Overall, the qPCR results of profile 4 illustrate the potential for rapidly identifying entire sets of related genes that are involved in iPSC differentiation using the nanodot platform. The gene expression changes correlated with improved differentiation confirmed the importance of genes, such as *IGFBP7* and *FN1*, conclusions that could be drawn only across several individual conventional investigations. Additionally, similar expression of genes, such as *SERPINE*, *MMP9*, and *KRT19*, indicates the possibility of more nuanced roles in differentiation than previously thought, warranting further examination. Furthermore, these results highlight the ability of the nanodot platform to provide physical stimulation during the iPSC differentiation. While iPSCs are routinely cultured on biocompatible scaffolds such as Matrigel that serve as rudimentary ECMs, the crucial biophysical and mechanical cues provided by the in vivo stem cell niche are not always recapitulated accurately or consistently, and the crucial regulation provided by ECM is therefore difficult to observe or control when using traditional differentiation protocols. Differentiation of iPSCs deliberately cultured on nanodot arrays revealed several noteworthy ECM-related gene expression trends that could be directly targeted to significantly improve the differentiation efficiency. Thus, the nanodot platform acts as a critical source of physical stimulation that enables the identification of key differentiation-related genes that would not be easily observed through conventional chemically induced differentiation alone.

Relative expression levels of 10 nanodot-correlated genes in profile 4 before and after treatment with either NSC-663284 (A) or BRD K98 (B) (*t*-test) (*p < 0.05, **p < 0.005, ***p < 0.0005, ****p < 0.0001). Red boxes indicate significant up- or downregulation by small molecule drug treatment.

**Figure 8:**
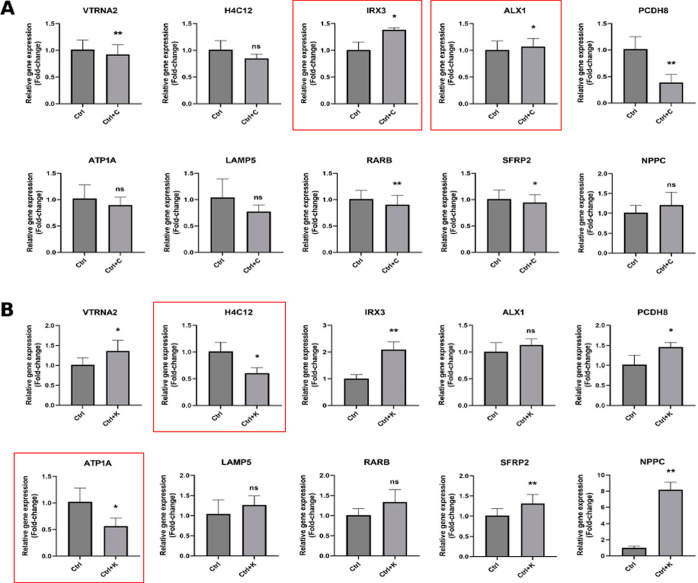
Cell growth-related genes of Profile 22 are significantly modulated by treatment with CMap-selected small-molecule drugs.

Although carmofur and KPT-330 were selected to oppositely regulate genes related to cell growth in profile 22, both treatment groups exhibited improved differentiation compared to the control. The gene expression levels of 10 highly significant genes of profile 22 before and after treatment are shown in Figure 8. One of the ten genes (*IRX3*) was significantly upregulated by carmofur (Figure 8A), while two of the ten genes (*H4C12*, *ATP1A*) were significantly downregulated by KPT-330 (Figure 8B). Interestingly, genes such as *PCDH8* demonstrated contrasting expression patterns between the two treatment groups. Protocadherin 8 (PCDH8) has been shown to inhibit the Wnt/β-catenin signaling pathway.^64^ Notably, Wnt/β-catenin signaling plays a pivotal role in regulating cardiogenesis in a biphasic manner, and Wnt inhibitors are essential components that tightly control the pathway throughout CM differentiation.^65^ In this study, despite both exhibiting markedly improved differentiation efficiency, cells treated with carmofur exhibited seemingly lower expression of PCDH8 while KPT-330-treated cells demonstrated significantly increased expression. These conflicting results underscore the need to further investigate the complex interactions among genes of profile 22.

Relative expression levels of 10 nanodot-correlated genes in profile 22 before and after treatment with either carmofur (A) or KPT-330 (B) (*t*-test) (*p < 0.05, **p < 0.005, ***p < 0.0005, ****p < 0.0001). Red boxes indicate significant up- or downregulation by small molecule drug treatment.

**Figure 9:**
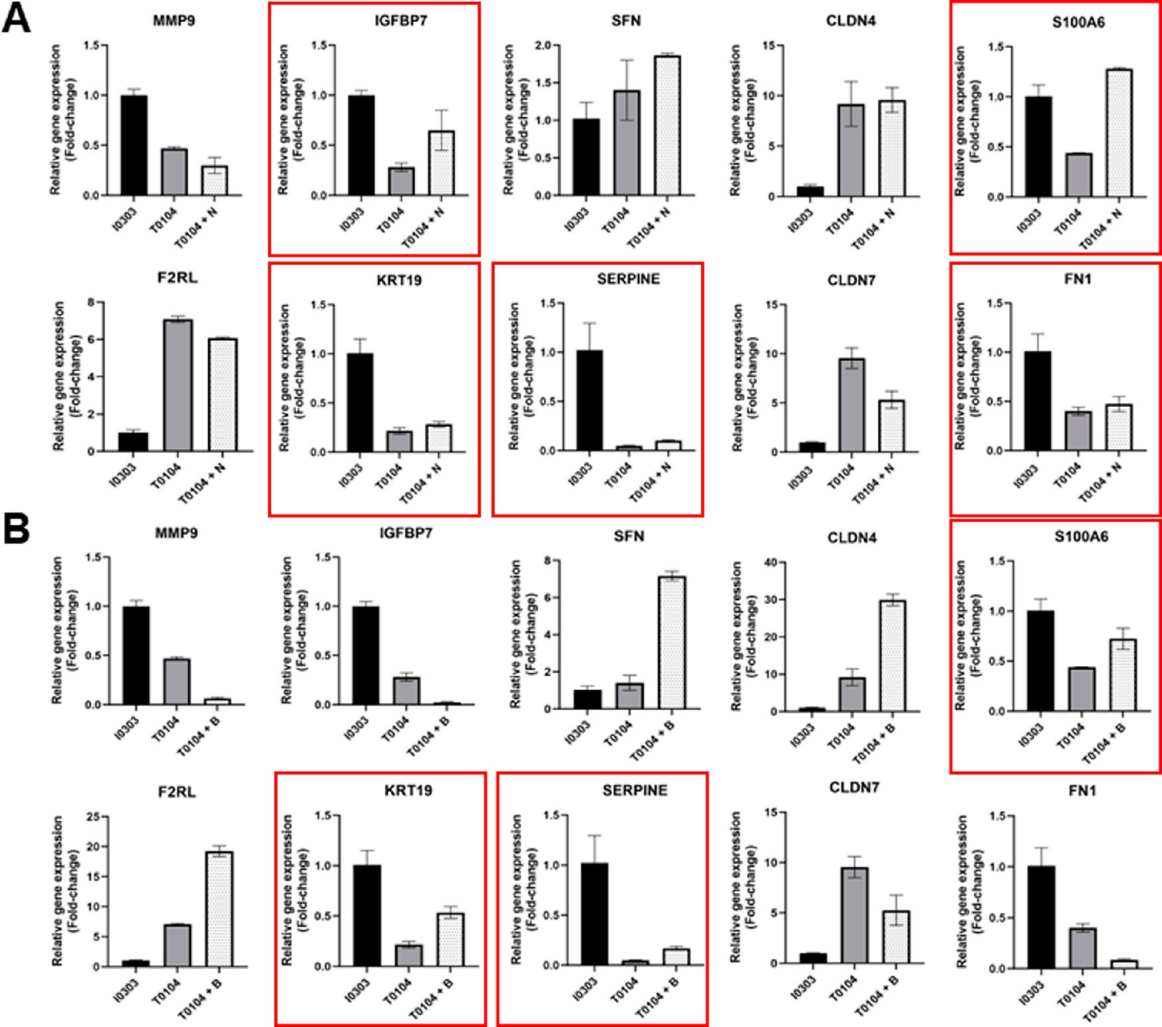
Expression of Profile 4 genes in T0104-derived CMs can be calibrated to match the expression profile of I0303-derived CMs.

**Figure 10:**
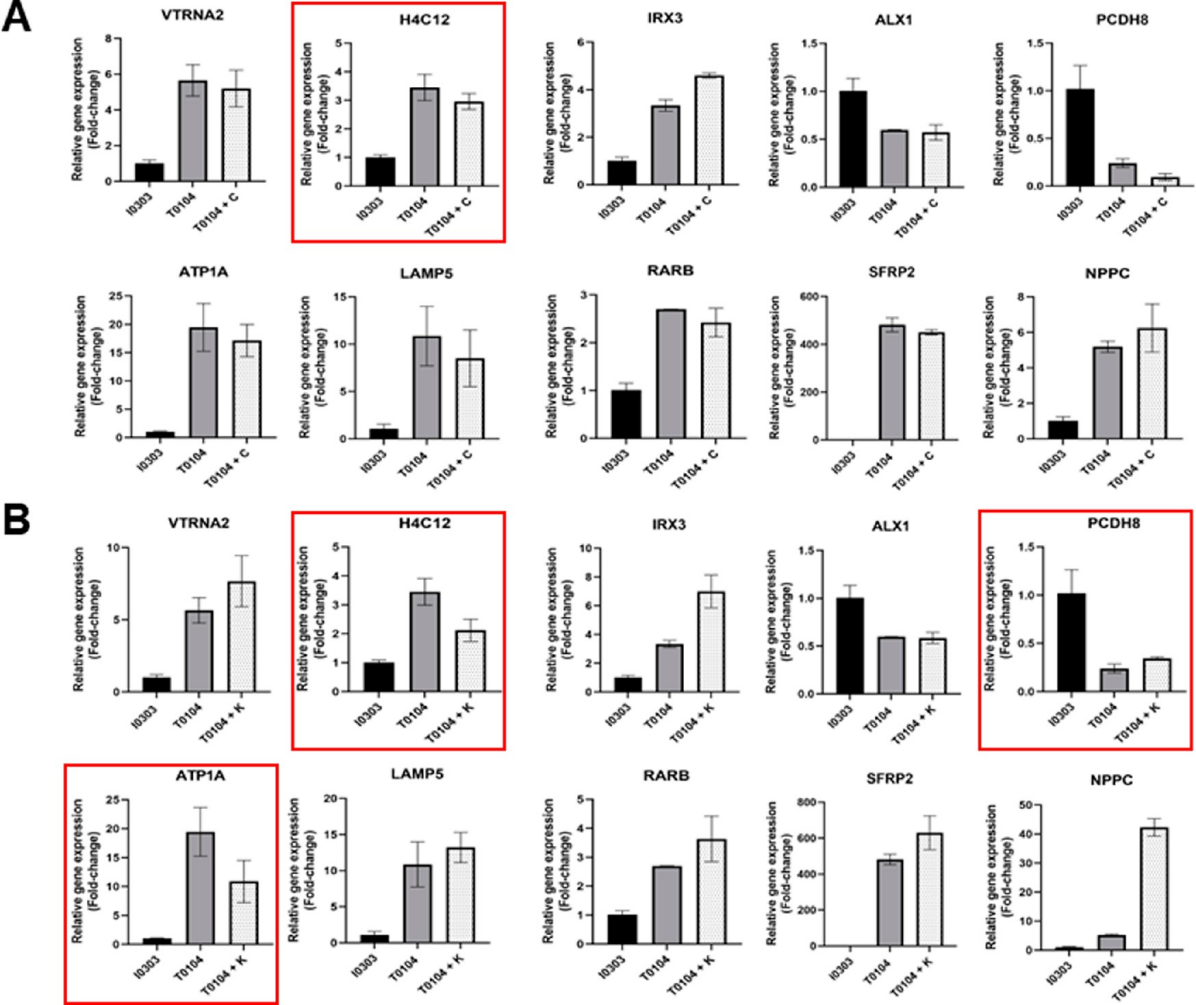
Small-molecule drug treatment results in differential expression of Profile 22 genes in T0104-derived CMs when compared to I0303-derived CMs.

To fully visualize the ability to shape iPSC expression profiles using the nanodot platform, CMs differentiated from line I0303, which possesses a naturally high differentiation propensity, were selected for comparison with CMs derived from T0104 cells, which initially showed poor differentiation efficiency. Figures 9 and 10 show the relative expression levels of the ten selected genes of profiles 4 and 22 in I0303-derived CMs, T0104-derived CMS before drug treatment, and T0104-derived CMs after drug treatment.

Following treatment with NSC 663284 (Figure 8A), five of the ten profile 4 genes (*IGFB7*, *S100A6*, *F2RL*, *KRT19*, and *SERPINE*) were calibrated to show expression levels closer to differentiated I0303 CMs, leading to significantly improved CM differentiation. Before treatment, T0104 CMs showed not only significantly divergent expression of profile 4 genes when compared to I0303 CMs but also drastically lower differentiation potential. The results in Figure 9A demonstrate that the innate differences in differentiation propensity, likely caused by inherently contrasting gene expression, could be overcome by selecting a drug that encourages a gene expression profile more akin to that of a cell line with a high differentiation potential such as I0303. On the other hand, only three of the ten genes (*S100A6*, *KRT19*, and *SERPINE*) exhibited expression levels closer to I0303 CMs after treatment with BRD K98 (Figure 9B), and differentiation efficiency remained poor. Aside from the three profile 4 genes that were similarly regulated by both drugs (*S100A6*, *KRT19*, and *SERPINE*), expression levels of both *IGFBP7* and *F2RL* were tuned to be more closely aligned with levels in I0303 CMs after treatment with NSC 663284 but not with BRD K98. While *IGFBP7* is known to play a role in CM differentiation, these results also suggest the possibility that *F2RL* is involved in the differentiation process as well. Though protease-activated receptor 1 (PAR1), encoded by the *F2RL* gene, has been shown to induce heart remodeling via activation of the ERK pathway,^66^ its influence on CM differentiation is unclear. Nevertheless, the contrasting expression levels of *F2RL* between well differentiated and poorly differentiated CMs suggests potential involvement, especially considering the critical role of the ERK pathway in CM differentiation.^67^

Relative expression levels of 10 nanodot-correlated profile 4 genes in I0303-derived CMs as well as T0104-derived CMs before and after treatment with either NSC 663284 (A) or BRD K98 (B) Red boxes indicate significant genes whose expression was significantly modulated to resemble I0303 levels more closely by small-molecule drug treatment.

Interestingly, while both carmofur and KPT-330 treatment resulted in improved CM differentiation, the expression of profile 22 genes was generally not calibrated to match I0303 in either group. Specifically, in carmofur-treated iPSCs, expression of only one gene (*H4C12*) more closely resembled levels in I0303 (Figure 10A), and in cells treated with KPT-330, expression of only three genes (*H4C12*, *PCDH8*, and *ATP1A*) shifted toward I0303 levels (Figure 10B). Notably, the expression of both *IRX3* and *NPPC* significantly increased following treatment with either carmofur or KPT-330, in contrast to the low expression levels observed in I0303 CMs. *IRX3* is known to maintain proper electrical propagation of heart ventricles by regulating transcription of several gap junction genes.^68^ Natriuretic peptide C encoded by *NPPC* is expressed primarily in the heart, where it modulates cyclic guanosine monophosphate (cGMP)-dependent signaling cascade to regulate cardiac remodeling.^69^ Though neither *IRX3* nor *NPPC* has been directly linked to CM differentiation, their significant upregulation coincided with improved differentiation efficiency in both carmofur and KPT-330 treatment groups, suggesting possible involvement in differentiation. More importantly, the contrast in expression of profile 22 genes such as *IRX3* and *NPPC* between iPSCs treated with small molecule drugs and I0303-derived CMs confirms multiple avenues for improving CM differentiation. While iPSCs that showed an expression profile more similar to I0303 exhibited an elevated differentiation ability, iPSCs that showed contrasting profiles following drug treatment also demonstrated improved differentiation; in other words, the nanodot platform was able to reveal multiple gene expression profiles that may uniquely contribute to CM differentiation.

Relative expression levels of 10 nanodot-correlated profile 22 genes in I0303-derived CMs as well as T0104-derived CMs before and after treatment with either carmofur (A) or KPT-330 (B) Red boxes indicate significant genes whose expression was significantly modulated to resemble I0303 levels more closely by small-molecule drug treatment.

Together, the results of Figures 9 and 10 highlight the variability of different iPSCs and the versatility of the nanodot platform. The process of differentiation is governed by an incredibly intricate network of different pathways that can be further confounded by variation stemming from factors, such as cell origin and epigenetic modifications. Thus, studying and optimizing differentiation requires a multifaceted approach, which the nanodot platform fulfills. The ability to observe an ensemble of differentiation related changes in gene expression allows for the identification of well-defined gene sets that, when combined with CMap, facilitate rapid screening and testing of potential differentiation enhancing drugs. Furthermore, while iPSCs of different origin possess varying differentiation capacity due to innate characteristics, the unique expression profile of any individual cell line can be revealed and targeted with existing drugs. Repurposed drugs with differentiation-improving capabilities such as NSC 663284 can be matched to certain expression profiles, while other drugs may be repurposed to treat other profiles with different expression patterns more effectively. As more profiles are analyzed and more drugs are screened and tested, the process of improving differentiation efficiency is expected to become both more personalized and more streamlined. Overall, the proposed nanodot platform is a useful tool that enables rapid drug screening to guide future investigation of the differentiation process, and the framework described presents a promising holistic approach toward deciphering iPSC differentiation for personalized clinical applications.

## 4 Conclusions

In this study, we combined a chemical-based iPSC differentiation protocol with a nanodot platform that acts as an artificial stem cell niche to enable study of iPSC differentiation in greater detail. Directed differentiation of iPSCs cultured on nanodot arrays revealed gene expression trends that seemingly hindered CM differentiation. The observed expression patterns allowed for the selection of small-molecule drugs via CMap that could effectively modulate the CM differentiation efficiency. Most notably, qPCR analysis of drug-induced gene expression changes revealed both expected and unexpected trends in various differentiation-related genes, validating the utility of the platform as a powerful tool for studying differentiation. Taken together, our results confirm the possibility of elucidating the complex mechanisms behind iPSC differentiation by leveraging the nanodot platform. This research serves as a preliminary step toward optimizing iPSC technology for clinical applications.

## Supporting Information Available

Nanodot characterization, GO term analysis results, STEM analysis results, and CMap ranking results (PDF)
Spontaneous beating observed in a small clump of cells after 21 days (AVI)
BRD K98-treated cells exhibiting no beating at all after 21 days of differentiation (AVI)
Beating observed in a much larger area of cell clumps (AVI)
Larger area of cell clumps demonstrated beating after treatment with carmofur (AVI)
Majority of cell clumps treated with KPT-330 exhibited beating (AVI)

## Supplementary Material

am4c04451_si_001.pdf

am4c04451_si_002.avi

am4c04451_si_003.avi

am4c04451_si_004.avi

am4c04451_si_005.avi

am4c04451_si_006.avi
