# Supplementary material for: Improving iPSC Differentiation Using a Nanodot Platform
Source: ACS Appl Mater Interfaces. 2024 Jul 1;16(28):36030–46. doi: 10.1021/acsami.4c04451 (PMC11261571; doi:10.1021/acsami.4c04451)
Supplement: Supplementary file 1 — am4c04451_si_001.pdf [file am4c04451_si_001.pdf]

Supporting Information

Improving iPSC differentiation using a nanodot platform

Men Yee Chiew [a, b, ‡], Erick Wang [b, c, ‡], Kuan-Chun Lan [d], Yan-Ren Lin [e, f, g, h], Yu-Huan Hsueh [i, j], Yuan-Kun Tu [j], Chu-Feng Liu [k, l], Po-Chun Chen [m, *], Huai-En Lu [a, n, o, *], Wen Liang Chen [a, b, c, o, *]

a. Center for Regenerative Medicine and Cellular Therapy, National Yang Ming Chiao Tung University, 300, Taiwan, ROC
b. Department of Biological Science and Technology, National Yang Ming Chiao Tung University, Hsinchu, 300, Taiwan, ROC
c. College of Biological Science and Technology Industrial Ph.D. Program, National Yang Ming Chiao Tung University, Hsinchu, 300, Taiwan, ROC
d. Center for iPS Cell Research and Application (CiRA), Kyoto University, Kyoto, 606-8397, Japan
e. Department of Emergency and Critical Care Medicine, Changhua Christian Hospital, Changhua, 500, Taiwan, ROC
f. Department of Post Baccalaureate Medicine, College of Medicine, National Chung Hsing University, Taichung, 402, Taiwan, ROC
g. School of Medicine, Kaohsiung Medical University, Kaohsiung, 807, Taiwan, ROC
h. School of Medicine, Chung Shan Medical University, Taichung, 402, Taiwan, ROC
i. College of Biological Science and Technology, National Yang Ming Chiao Tung University, 300, Taiwan, ROC
j. Department of Orthopedic Surgery, E-Da Hospital, I-Shou University, Kaohsiung, 824, Taiwan

k. Emergency Medicine Department, Kaohsiung Chang Gung Memorial Hospital, 833, Taiwan, ROC
l. Ph.D. Degree Program of Biomedical Science and Engineering, National Yang Ming Chiao Tung University, Hsinchu, 300, Taiwan, ROC
m. Institute of Materials Science and Engineering, National Taipei University of Technology, Taipei, 106, Taiwan, ROC
n. Institute of Biochemistry and Molecular Biology, National Yang Ming Chiao Tung University, 300, Taiwan, ROC
o. Bioresource Collection and Research Center, Food Industry Research and Development Institute, Hsinchu City, 300, Taiwan, ROC

***Corresponding Authors:**

Wen-Liang Chen, Ph.D., Professor

Department of Biological Science and Technology, National Yang Ming Chiao Tung University, 75 Bo'ai St, Hsinchu, Taiwan, ROC

Phone: +886-3-5712121 ext. 59711

Email address: wenurea@yahoo.com.tw

Huai-En Lu, Ph.D.

Center for Regenerative Medicine and Cellular Therapy, National Yang Ming Chiao Tung University, 75 Bo'ai St, Hsinchu, Taiwan, ROC

Phone: +886-3-5712121 ext. 59780

Email address: helu@nycu.edu.tw

Po-Chun Chen, Ph.D., Professor

Institute of Materials Science and Engineering, National Taipei University of Technology, 1 Zhongxiao E Rd, Section 3, Da’an District, Taipei, Taiwan, ROC

Phone: +886-2-27712171

Email: cpc@ntut.edu.tw

Figure S1

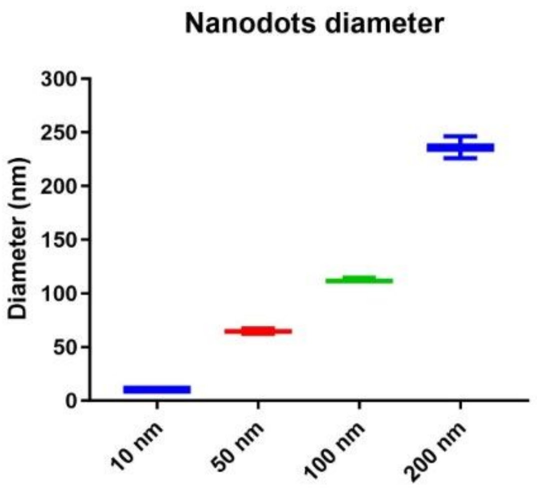

40

Figure S1. Diameter of tantalum oxide nanodots. The plot shows the representative measurement of nanodot size from random picked samples (n=6 from each group).

Figure S2

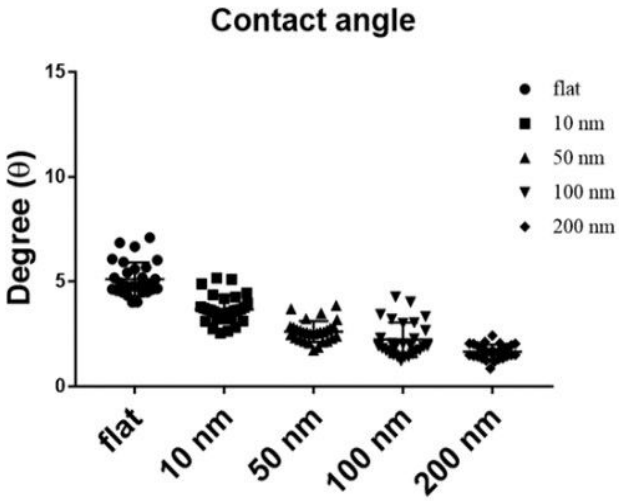

Figure S2. Wettability of tantalum oxide nanodots was measured using water contact angle. A total of 30 samples (n=30) from each group were selected for statistical analysis.

Figure S3

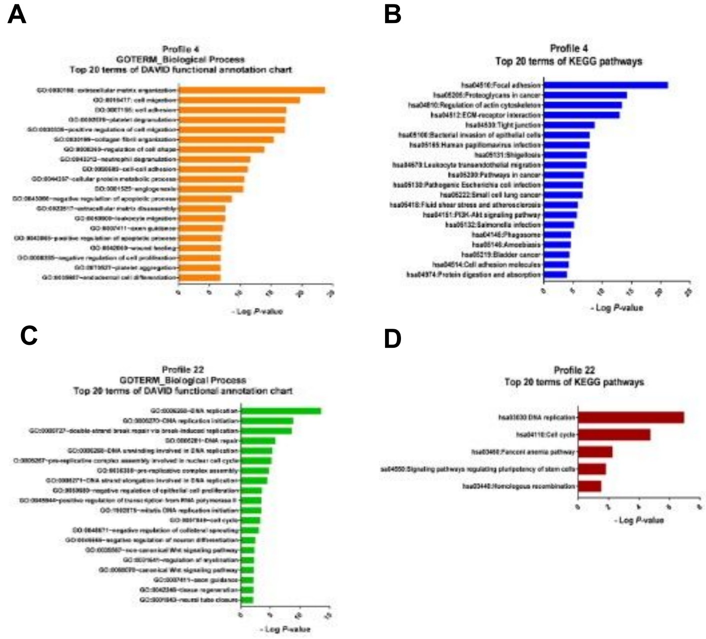

Figure S3. Gene Ontology term enrichment for genes in profiles 4 and 22.

Figure S4

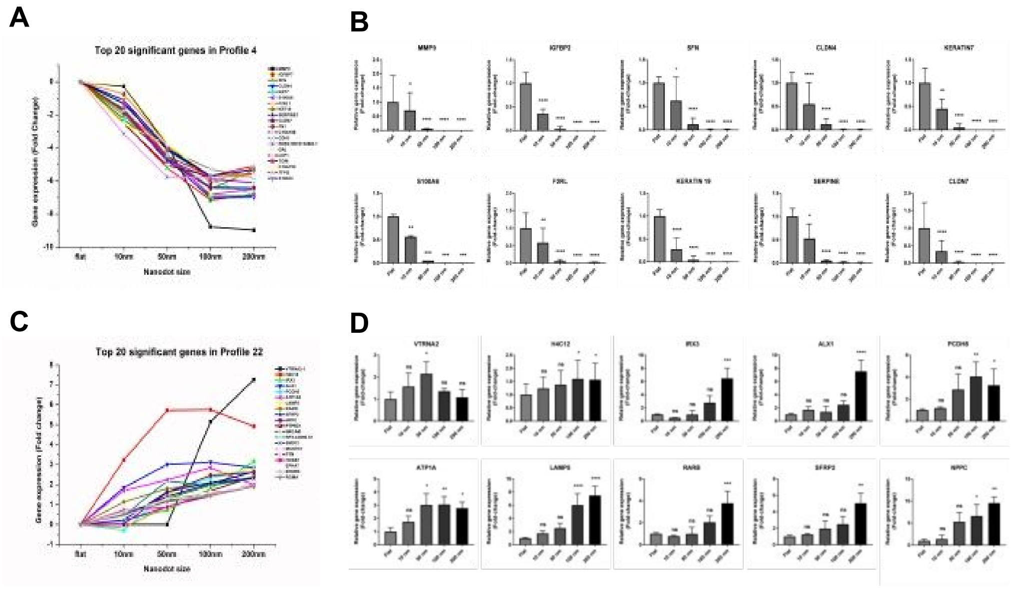

Figure S4. Relative expression of genes most highly correlated with increase in nanodot diameter. (A) Relative fold change of top 20 significant genes of profile 4 across different nanodot diameters. (B). qPCR of top 10 genes in profile 4 across different nanodot diameters. (C) Relative fold change of top 20 significant genes of profile 22 across different nanodot diameters. (D) qPCR of top 10 genes in profile 22 across different nanodot diameters.

Table S1: Taqman® Gene Expression Assay and their ID.

Gene	Gene Name	Assay ID
GAPDH	Glyceraldehyde-3-phosphate Dehydrogenase	Hs02758991_g1
MMP9	Matrix Metallopeptidase 9	Hs00957562_m1
IGFBP7	Insulin Like Growth Factor Binding Protein 7	Hs00266026_m1
SFN	Stratifin	Hs00968567_s1
CLDN4	Claudin 4	Hs00976831_s1
KRT7	Keratin 7	Hs00559840_m1
S100A6	S100 Calcium Binding Protein A6	Hs00170953_m1
F2RL1	F2R Like Trypsin Receptor 1	Hs00608346_m1
KRT19	Keratin 19	Hs01051611_gH
SERPINE1	Serpin Family E Member 1	Hs00167155_m1
CLDN7	Claudin 7	Hs00600772_m1
VTRNA2-1	Vault RNA 2-1	Hs04273370_s1
H4C12	H4 Clustered Histone 12	Hs03406440_gH
IRX3	Iroquois Homeobox 3	Hs01124217_g1
ALX1	ALX Homeobox 1	Hs01058467_m1
PCDH8	Protocadherin 8	Hs00159910_m1
ATP1A2	ATPase Na+/K+ Transporting Subunit Alpha 2	Hs00265131_m1
LAMP5	Lysosomal Associated Membrane Protein Family	Hs00202136_m1
RARB	Retinoic Acid Receptor Beta	Hs00977140_m1
SFRP2	Secreted Frizzled Related Protein 2	Hs01564480_m1
NPPC	Natriuretic Peptide C	Hs00360930_g1
NPPC	Natriuretic Peptide C	Hs00360930_g1
MYH7	myosin, heavy chain 7, cardiac muscle, beta	Hs01110632_m1
MYH6	myosin heavy chain 6	Hs01101425_m1
TNNT2	troponin T2, cardiac type	Hs00165960_m1
RYR2	ryanodine receptor 2	Hs00181461_m1
SCN5A	sodium voltage-gated channel alpha subunit 5	Hs00165693_m1
GJA1	gap junction protein alpha 1	Hs00748445_s1

Table S1. List of genes analyzed using qPCR.

Table S2: Top 100 gene ranking of Profile 4.

Rank	Gene Symbol	flat	10nm	50nm	100nm	200nm	max-min
1	MMP9	0	-0.27	-3.81	-8.77	-8.98	8.98
2	IGFBP7	0	-1.08	-4.45	-7.17	-6.8	7.17
3	SFN	0	-2.48	-5.2	-7.15	-6.81	7.15
4	CLDN4	0	-1.65	-4.66	-7.02	-6.98	7.02
5	KRT7	0	-2.26	-4.4	-6.92	-6.89	6.92
6	S100A6	0	-1.35	-4.13	-5.94	-6.85	6.85
7	F2RL1	0	-1.09	-4.55	-6.81	-6.5	6.81
8	KRT19	0	-2.01	-4.59	-6.48	-5.35	6.48
9	SERPINE1	0	-1.82	-4.8	-6.41	-6.46	6.46
10	CLDN7	0	-1.64	-4.58	-6.35	-6.41	6.41
11	FN1	0	-0.73	-3.79	-6.11	-5.19	6.11
12	C15orf48	0	-1.78	-4.69	-5.88	-6.09	6.09
13	CDH1	0	-1.78	-4.05	-5.28	-5.81	5.81
14	ENSG10010134880.1	0	-3.14	-5.75	-5.75	-5.75	5.75
15	GAL	0	-0.19	-3.69	-5.73	-5.56	5.73
16	LCP1	0	-2.52	-5.05	-5.73	-5.59	5.73
17	TCIM	0	-1.3	-4.11	-5.71	-5.3	5.71
18	C19orf33	0	-2.55	-3.78	-5.71	-5.71	5.71
19	TFPI2	0	-1.74	-4.48	-5.7	-5.09	5.7
20	S100A3	0	-1.1	-3.99	-5.7	-5.32	5.7
21	PLP2	0	-1.82	-4.59	-5.45	-5.68	5.68
22	CST6	0	-1.28	-4.57	-5.63	-5.21	5.63
23	CLDN6	0	-1.8	-3.88	-5.23	-5.57	5.57
24	UCA1	0	-0.99	-3.67	-5.43	-5.57	5.57
25	IL32	0	-1.8	-3.66	-4.86	-5.55	5.55
26	LAMC2	0	-2	-4.81	-5.55	-5.43	5.55
27	CDKN2B	0	-1.21	-4.21	-5.5	-5.38	5.5
28	MIR4435-2HG	0	-0.53	-2.67	-4.3	-5.45	5.45
29	CAPG	0	-1.25	-3.79	-5.45	-5.43	5.45
30	CDH3	0	-1.06	-3.49	-5.42	-4.67	5.42
31	TINAGL1	0	-1.67	-3.99	-4.61	-5.32	5.32
32	SPP1	0	-0.03	-2.29	-4.73	-5.31	5.31
33	ANXA2	0	-1.37	-3.16	-4.71	-5.3	5.3
34	COL6A3	0	-0.43	-3.85	-5.26	-4.49	5.26
35	ANXA3	0	-2.07	-2.57	-4.08	-5.25	5.25
36	TRBC2	0	-0.71	-3.92	-5.22	-5.25	5.25
37	CALB2	0	-2.03	-4.49	-5.18	-4.84	5.18
38	RAB25	0	-1.91	-4.09	-5.17	-4.72	5.17
39	GPR87	0	-1.09	-4.22	-5.15	-5.06	5.15
40	CD44	0	-0.93	-4.23	-5.14	-4.97	5.14
41	S100A11	0	-1.14	-3.31	-4.93	-5.07	5.07
42	CD9	0	-1.58	-3.74	-5.07	-4.34	5.07
43	PLAU	0	-0.15	-2.88	-4.49	-5.07	5.07
44	BHLHE40	0	-1.32	-3.99	-5.03	-4.87	5.03
45	S100A16	0	-1.06	-3.7	-4.62	-5.02	5.02
46	CAV1	0	-1.97	-3.89	-5.02	-4.55	5.02
47	ACTA1	0	-2.9	-4.21	-4.98	-4.73	4.98
48	EPCAM	0	-1.04	-3.76	-4.98	-4.66	4.98
49	EMP1	0	-0.7	-2.9	-4.91	-4.48	4.91
50	DMKN	0	-1.4	-3.12	-4.88	-4.35	4.88
51	LYPD1	0	-0.56	-3.2	-4.76	-4.24	4.76
52	NPPB	0	-3.05	-3.39	-4.72	-4.49	4.72
53	CYTOR	0	-1.19	-2.73	-4.33	-4.71	4.71
54	COL1A1	0	-0.39	-2.18	-4.53	-4.66	4.66
55	ACTC1	0	-1.44	-1.56	-3.12	-4.64	4.64
56	SPINT1	0	-1.14	-3.5	-4.48	-4.61	4.61
57	SERPINE2	0	-0.7	-3.02	-4.49	-4.57	4.57
58	CCL2	0	-0.17	-2.23	-4.35	-4.56	4.56
59	LMCD1	0	-2.55	-3.69	-4.38	-4.49	4.49
60	ITGA3	0	-0.64	-3.15	-4.24	-4.47	4.47
61	HSPB8	0	-1.26	-2.68	-4.43	-4.41	4.43
62	TAGLN2	0	-1.01	-2.77	-4.33	-4.42	4.42
63	ELN	0	-0.12	-3.38	-4.39	-3.96	4.39
64	PLPP4	0	-0.75	-3.3	-4.06	-4.39	4.39
65	EPS8L2	0	-1.54	-3.17	-4.06	-4.34	4.34
66	CAVIN1	0	-2.07	-2.72	-3.67	-4.24	4.24
67	KRT17	0	-2.45	-3.29	-4.24	-4.08	4.24
68	ANXA1	0	-1.11	-1.88	-2.97	-4.23	4.23
69	TSPAN2	0	-2.22	-3.74	-4.17	-4.11	4.17
70	PLAT	0	-0.67	-3.24	-4.12	-4.03	4.12
71	MIR205HG	0	-0.76	-2.31	-4.11	-4.12	4.12
72	PDGFA	0	-1.32	-3.18	-4.07	-3.72	4.07
73	COL1A2	0	-0.74	-2.15	-3.46	-4.03	4.03
74	MMP14	0	-0.2	-2.24	-3.98	-4.03	4.03
75	TNC	0	-1.07	-3.03	-3.23	-4.02	4.02
76	ARHGDIB	0	-0.97	-2.31	-3.64	-3.99	3.99
77	ANXA11	0	-1.01	-2.69	-3.35	-3.98	3.98
78	ANKRD1	0	-1.2	-1.98	-3.52	-3.96	3.96
79	CAVIN3	0	-1.21	-3.21	-3.93	-3.51	3.93
80	CD74	0	-0.87	-2.56	-3.01	-3.92	3.92
81	FBLIM1	0	-0.7	-2.41	-3.89	-3.63	3.89
82	JUNB	0	-0.44	-2.83	-3.73	-3.88	3.88
83	HSPB1	0	-1.05	-2.77	-3.86	-3.8	3.86
84	LBH	0	-1.24	-2.43	-3.82	-3.34	3.82
85	S100A10	0	-1.31	-3.1	-3.72	-3.81	3.81
86	BAMBI	0	-0.89	-3.14	-3.79	-3.28	3.79
87	STAP2	0	-1.21	-2.78	-3.34	-3.79	3.79
88	TAGLN	0	-1.3	-1.84	-3.16	-3.77	3.77
89	ACTG2	0	-0.97	-0.41	-2.1	-3.77	3.77
90	TNFRSF21	0	-1.13	-2.58	-3.6	-3.76	3.76
91	CDKN1A	0	-0.79	-2.59	-3.28	-3.72	3.72
92	PERP	0	-1.2	-3	-3.69	-3.42	3.69
93	IER3	0	-0.77	-2.34	-3.64	-3.63	3.64
94	COL9A3	0	-0.32	-2.57	-3.64	-3.48	3.64
95	CCDC80	0	-0.62	-2.01	-3.22	-3.63	3.63
96	PIEZO1	0	-1.04	-2.58	-3.44	-3.59	3.59
97	TGFB1	0	0.03	-2.21	-3.54	-3.01	3.57
98	DSG2	0	-1.36	-3.12	-3.55	-3.42	3.55
99	GSN	0	-0.36	-2.54	-3.46	-3.54	3.54
100	CNN1	0	-1.49	-1.93	-2.89	-3.54	3.54

Table S2. Top 100 profile 4 genes determined to be significantly correlated with nanodot diameter, as determined by STEM analysis.

Table S3Top 100 gene ranking of Profile 22 .

Rank	Gene Symbol	flat	10nm	50nm	100nm	200nm	max-min
1	VTRNA2-1	0	0	0	5.15	7.26	7.26
2	H4C12	0	3.24	5.72	5.75	4.92	5.75
3	IRX3	0	0.22	0.72	1.79	3.16	3.16
4	ALX1	0	1.85	3.01	3.12	2.85	3.12
5	PCDH8	0	-0.29	1.6	2.33	2.78	3.07
6	ATP1A2	0	1.7	2.24	2.85	1.99	2.85
7	LAMP5	0	0.45	1.49	2.54	2.75	2.75
8	RARB	0	1.13	1.8	2.06	2.65	2.65
9	SFRP2	0	0.48	1.4	2.05	2.64	2.64
10	NPPC	0	0.02	1.61	2.46	2.61	2.61
11	PTPRZ1	0	0.47	1.17	1.69	2.41	2.41
12	NRCAM	0	0.4	1.29	1.97	2.39	2.39
13	RP3-430N8.10	0	0.45	2.18	1.95	2.32	2.32
14	SMOC1	0	0.23	1.64	2.11	2.3	2.3
15	MUSTN1	0	-0.15	0.91	1.41	1.99	2.14
16	PTN	0	0.19	0.89	1.46	1.96	1.96
17	HOXB1	0	0.72	0.86	1.57	1.96	1.96
18	EPHA7	0	0.39	1.29	1.64	1.9	1.9
19	EDNRB	0	0.66	1.26	1.48	1.9	1.9
20	RGMA	0	0.51	1.13	1.45	1.89	1.89
21	C1orf194	0	1.25	1.15	1.37	1.89	1.89
22	DLK1	0	-0.05	0.58	1.56	1.7	1.75
23	FAM117A	0	0.17	1.02	1.52	1.72	1.72
24	MSH5	0	1.06	0.93	1.12	1.7	1.7
25	RP11-339B21.12	0	-0.01	0.28	0.72	1.66	1.67
26	SOX2	0	0.28	0.91	1.3	1.66	1.66
27	LRIG1	0	0.35	1	1.41	1.66	1.66
28	ARL4A	0	-0.25	0.53	0.95	1.37	1.62
29	FRZB	0	1.38	1.6	1.62	1.57	1.62
30	CRNDE	0	0.32	0.57	0.86	1.61	1.61
31	CA14	0	0.5	0.88	1.08	1.58	1.58
32	PLP1	0	0.06	0.76	1.52	1.34	1.52
33	RP11-631N16.4	0	0.27	0.94	1.03	1.48	1.48
34	SKIDA1	0	0.48	0.92	1.32	1.48	1.48
35	PAX3	0	0.39	0.81	1.06	1.47	1.47
36	GPHN	0	1.04	0.79	1.24	1.42	1.42
37	C6orf118	0	0.31	0.86	1.08	1.42	1.42
38	NR6A1	0	0.3	0.8	1.2	1.4	1.4
39	ROBO3	0	0.2	1.13	1.37	1.28	1.37
40	MYCN	0	0.78	0.94	1.36	1.32	1.36
41	IFT57	0	0.29	1.04	1.33	1.34	1.34
42	BOC	0	0.58	0.73	0.84	1.32	1.32
43	MEIS1	0	0.59	0.8	0.82	1.31	1.31
44	SAPCD1	0	1.06	1.14	1.29	1.25	1.29
45	FANCI	0	0.64	1.01	1.26	0.95	1.26
46	POLD1	0	0.85	1.05	1.26	1.19	1.26
47	RRNAD1	0	0.34	0.88	0.94	1.26	1.26
48	ACAT2	0	0.55	1.03	1.25	1.25	1.25
49	PAN2	0	0.54	1.25	1.23	0.97	1.25
50	MAP2K5	0	0.43	0.61	0.51	1.25	1.25

Rank	Gene Symbol	flat	10nm	50nm	100nm	200nm	max-min
51	UNG	0	0.6	0.88	1.04	1.24	1.24
52	SLC25A27	0	0.29	0.64	1	1.24	1.24
53	HOXA2	0	0.39	0.65	0.91	1.23	1.23
54	HOTAIRM1	0	0.33	0.42	0.69	1.22	1.22
55	SARS2	0	0.71	0.89	0.88	1.22	1.22
56	RP11-159D12.5	0	0.65	0.79	0.9	1.22	1.22
57	PRTG	0	0.05	0.78	0.96	1.21	1.21
58	DYNC2H1	0	0.31	0.58	0.91	1.21	1.21
59	DEPDC1B	0	0.49	1.06	1.04	1.2	1.2
60	FIRRE	0	0.63	0.55	0.94	1.2	1.2
61	BRCA1	0	0.38	1.2	1.11	1.05	1.2
62	ORC1	0	0.66	1.19	1.17	1.03	1.19
63	CDC7	0	0.49	1.09	1.03	1.17	1.17
64	CCDC160	0	0.25	0.66	0.98	1.16	1.16
65	GREB1	0	0.58	1.04	1.16	1.08	1.16
66	CSKMT	0	0.86	0.74	0.83	1.16	1.16
67	MIR17HG	0	0.65	0.89	0.96	1.15	1.15
68	CDCA7	0	0.66	1.1	1.15	1.14	1.15
69	GAS1	0	0.25	0.92	0.97	1.15	1.15
70	TRIM71	0	0.31	0.88	1.15	1.14	1.15
71	MIR124-2HG	0	0.02	0.22	0.57	1.15	1.15
72	SLC27A5	0	0.42	0.71	1.15	0.76	1.15
73	FGFR2	0	-0.1	0.2	0.39	1.03	1.13
74	KIF15	0	0.53	0.77	0.86	1.13	1.13
75	TMEM97	0	0.52	0.86	1.12	1.02	1.12
76	FGF13	0	0.12	0.93	1.12	1.1	1.12
77	TMEM47	0	0.14	0.46	0.69	1.12	1.12
78	ALPL	0	0.26	0.75	1.12	1.09	1.12
79	ZBTB12	0	0.59	0.88	0.9	1.12	1.12
80	NEFM	0	1.11	1.05	1.11	0.98	1.11
81	RAD51AP1	0	0.46	1.1	0.95	1.11	1.11
82	LIN28A	0	0.25	0.85	1.1	1.06	1.1
83	DDX11	0	0.88	1.03	0.92	1.1	1.1
84	PPP1R1A	0	0.39	0.46	0.92	1.09	1.09
85	NCAPH	0	0.91	1.03	1	1.08	1.08
86	ZEB2	0	0.37	0.78	1	1.08	1.08
87	MND1	0	0.84	0.87	0.89	1.07	1.07
88	FAM110B	0	0.66	0.81	1.07	0.8	1.07
89	CDT1	0	0.94	0.81	0.95	1.07	1.07
90	LMNB1	0	0.71	1.04	1.06	1.04	1.06
91	PCLAF	0	0.59	1.06	0.94	1.03	1.06
92	MCM5	0	0.38	0.91	1.06	0.72	1.06
93	NELL2	0	0.31	0.82	0.83	1.06	1.06
94	IFT74	0	0.64	0.97	1.06	1.01	1.06
95	MCM4	0	0.51	0.96	0.93	1.05	1.05
96	GINS2	0	0.5	0.86	1.05	0.94	1.05
97	RMI2	0	0.45	0.72	0.99	1.05	1.05
98	GREB1L	0	0.31	0.62	0.92	1.05	1.05
99	MCM3	0	0.56	0.95	1.04	1.04	1.04
100	SMIM19	0	0.48	0.49	0.58	1.04	1.04

Table S3. Top 100 profile 22 genes determined to be significantly correlated with nanodot diameter, as determined by STEM analysis.

Table S4: Top 20 candidates of chemicals that have opposite effects with Profile 4.

Perturbagen	Mechanism of Action	Target (s)	Normalized cs
BRD-K24418561	N/A	N/A	2.55
NSC-663284	CDC inhibitor	CDC25A|CDC25B|CDC25C	2.55
BRD-K99636700	N/A	N/A	2.52
BRD-K32040515	N/A	N/A	2.5
BRD-K06748700	N/A	N/A	2.47
BRD-K98824517	N/A	N/A	2.47
SA-247615	N/A	N/A	2.45
BRD-K06765193	N/A	N/A	2.45
QW-BI-011	Histone lysine methyltransferase inhibitor	EHMT2	2.45
SB-225002	CC chemokine receptor antagonist	CXCR2	2.44
BRD-K09528868	N/A	N/A	2.44
TC-H-106	HDAC inhibitor	HDAC1|HDAC2|HDAC3|HDAC8	2.43
entinostat	HDAC inhibitor	HDAC1|HDAC2|HDAC3|HDAC9	2.41
cytochalasin-d	Tubulin inhibitor	ACTA1	2.41
BRD-K92722247	N/A	N/A	2.41
BRD-K06009608	N/A	N/A	2.41
vinorelbine	Tubulin inhibitor	N/A	2.41
BRD-K00007652	N/A	N/A	2.39
olmesartan-medoxomil	Angiotensin receptor antagonist	AGTR1	2.38
selumetinib	MEK inhibitor	MAP2K1|MAP2K2	2.38

Table S4. Top 20 small molecule drug candidates that regulate profile 4 genes in opposite direction compared to nanodots.

Table S5: Top 20 candidates of chemicals that have similar effects with Profile 4.

Perturbagen	Mechanism of Action	Target(s)	Normalized cs
BRD-K98645985	N/A	N/A	-2.62
BRD-K73008154	N/A	N/A	-2.54
QL-X-138	N/A	N/A	-2.53
BRD-K83694683	N/A	N/A	-2.46
brefeldin-a	N/A	N/A	-2.45
BRD-K98645985	N/A	N/A	-2.4
tanespimycin	N/A	N/A	-2.37
PHA-848125	CDK inhibitor|Growthfactor receptor inhibitor	CDK2|CDK4|NTRK1|CDK1|CDK5|CDK7	-2.37
BRD-K91583156	N/A	N/A	-2.36
BRD-K98645985	N/A	N/A	-2.36
D-4476	TGF-beta receptor inhibitor	CSNK1A1|CSNK1D|TGFB1	-2.32
LY-2157299	TGF-beta receptor inhibitor	TGFBR1	-2.29
A-443654	N/A	N/A	-2.29
GSK-1070916	Aurora kinase inhibitor	AURKB|AURKC|AURKA|CYP2D6|CYP3A4	-2.29
BRD-U08759356	EGFR inhibitor	N/A	-2.26
simeprevir	HCV inhibitor	CYP2C19|CYP2C8|SLCO1B3|CYP1A2|CYP3A4	-2.25
BRD-K98431828	N/A	N/A	-2.25
BRD-K67236950	N/A	N/A	-2.25
brefeldin-a	Protein synthesis inhibitor|BIG1 inhibitor	ARFGEF1|ARFGEF2|GBF1|ARF1|CYTH2	-2.24
BRD-K27451531	N/A	N/A	-2.24

Table S5. Top 20 small molecule drug candidates that regulate profile 4 genes in similar direction compared to nanodots.

Table S6: Top 20 candidates of chemicals that have similar effects with Profile 22.

Perturbagen	Mechanism of action	Target(s)	Normalized cs
carmofur	Thymidylate synthase inhibitor	TYMS	2.43
roscovitine	CDK inhibitor	CDK2|CDK9|CDK7|CDK1|CDK5	2.2
BRD-A68721828	N/A	N/A	2.19
PF-750	FAAH inhibitor	FAAH	2.18
BRD-A49765801	Glucocorticoid receptor agonist	NR3C1|SERPINA6	2.16
gefitinib	EGFR inhibitor	EGFR|CYP2C19	2.15
monastrol	Kinesin inhibitor	KIF11	2.15
fmk	N/A	N/A	2.15
BRD-K80672993	Pyruvate kinase isozyme activator	N/A	2.15
BRD-K67808578	N/A	N/A	2.14
BRD-K04779531	N/A	N/A	2.14
LY-456236	Glutamate receptor antagonist	GRM1	2.13
BRD-K55575531	N/A	N/A	2.12
mitomycin-c	DNA alkylating agent|DNA inhibitor	N/A	2.12
BRD-K80672993	Pyruvate kinase isozyme activator	N/A	2.11
SN-38	Topoisomerase inhibitor	TOP1	2.09
BRD-K60932973	N/A	N/A	2.09
BRD-K31841256	N/A	N/A	2.09
pyrimethamine	Dihydrofolate reductase inhibitor	DHFRP1|HEXA|STAT3|DHFR|SLC47A1	2.09
levocabastine	Histamine receptor antagonist	HRH1|NTSR2	2.09

Table S6. Top 20 small molecule drug candidates that regulate profile 22 genes in similar direction compared to nanodots.

Table S7Top 20 candidates of chemicals that have opposite effects with Profile 22.

Perturbagen	Mechanism of action	Target(s)	Normalized cs
KPT-330	Exportin antagonist	XPO1	-2.65
homosalate	HSP inducer	N/A	-2.64
BRD-K09907482	Tyrosine phosphatase inhibitor	N/A	-2.63
BRD-K86961442	N/A	N/A	-2.62
palbociclib	CDK inhibitor	CDK4|CDK6|CCND3	-2.61
BRD-K61195623	Tubulin inhibitor|Angiogenesisinhibitor|Apoptosis stimulant	N/A	-2.61
naproxol	Anti-inflammatory	PTGS1|PTGS2	-2.59
CD-1530	N/A	N/A	-2.58
mepacrine	NFKB inhibitor|Cytokine production inhibitor|p53 activato	TP53|AKT1|MTOR|NFKB1|PL A2G2A|PLA2G2D|PLA2G1B| PLA2G4A|PLA2G6|PLCL1	-2.56
selamectin	Nematocide	N/A	-2.56
danusertib	Aurora kinase inhibitor|FGFR inhibitor	AURKA|AURKB|AURKC|FGF R1|NTRK1|RET|BCR|SLK	-2.55
BRD-K15050703	N/A	N/A	-2.54
JWE-035	Aurora kinase inhibitor	AURKA	-2.53
BRD-K86961442	N/A	N/A	-2.52
BRD-K64645183	N/A	N/A	-2.51
FCCP	Mitochondrial oxidative phosphorylation uncoupler	N/A	-2.51
kinetin-riboside	Apoptosis stimulant	N/A	-2.51
AMG-232	MDM inhibitor	MDM2	-2.51
rottlerin	N/A	N/A	-2.51
AG-879	Tyrosine kinaseinhibitor|Angiogenesisinhibitor|VEGFR inhibitor	KDR|ERBB2|NTRK1	-2.5

Table S7. Top 20 small molecule drug candidates that regulate profile 22 genes in opposite direction compared to nanodots.
